# A Brewster route to Cherenkov detectors

**DOI:** 10.1038/s41467-021-25822-x

**Published:** 2021-09-21

**Authors:** Xiao Lin, Hao Hu, Sajan Easo, Yi Yang, Yichen Shen, Kezhen Yin, Michele Piero Blago, Ido Kaminer, Baile Zhang, Hongsheng Chen, John Joannopoulos, Marin Soljačić, Yu Luo

**Affiliations:** 1 Interdisciplinary Center for Quantum Information, State Key Laboratory of Modern Optical Instrumentation, ZJU-Hangzhou Global Science and Technology Innovation Center, College of Information Science and Electronic Engineering, Zhejiang University, Hangzhou, 310027 China; 2 International Joint Innovation Center, ZJU-UIUC Institute, Zhejiang University, Haining, 314400 China; 3 School of Electrical and Electronic Engineering, Nanyang Technological University, Nanyang Avenue, Singapore, 639798 Singapore; 4 Particle Physics Department, Rutherford-Appleton Laboratory (STFC), Didcot, OX110QX UK; 5 Research Laboratory of Electronics and Department of Physics, Massachusetts Institute of Technology, Cambridge, MA 02139 USA; 6 Lightelligence, 268 Summer Street, Boston, MA 02210 USA; 7 Mantaline Corporation, 4754 E High Street, Mantua, OH 44106 USA; 8 European Organization for Nuclear Research (CERN), Geneva, 1211 Switzerland; 9 Cavendish Laboratory, University of Cambridge, Cambridge, CB3 0HE UK; 10 Department of Electrical Engineering, Technion-Israel Institute of Technology, Haifa, 32000 Israel; 11 Division of Physics and Applied Physics, School of Physical and Mathematical Sciences, Nanyang Technological University, 21 Nanyang Link, Singapore, 637371 Singapore; 12 Centre for Disruptive Photonic Technologies, Nanyang Technological University, Singapore, 637371 Singapore

**Keywords:** Metamaterials, Photonic crystals, Imaging and sensing, Nanophotonics and plasmonics, Experimental particle physics

## Abstract

Cherenkov detectors enable a valuable tool to identify high-energy particles. However, their sensitivity and momentum coverage are limited by the refractive index of host materials. Especially, identifying particles with energy above multiple gigaelectronvolts requires host materials with a near-unity refractive index, which are limited to bulky gas chambers. Overcoming this fundamental material limit is important for future particle detectors yet remains a long-standing challenge. Here, we propose a different paradigm for Cherenkov detectors that utilizes the broadband angular filter made from stacks of variable one-dimensional photonic crystals. Owing to the Brewster effect, the angular filter is transparent only to Cherenkov photons from a precise incident angle. Particle identification is achieved by mapping each Cherenkov angle to the peak-intensity position of transmitted photons in the detection plane. Such angular filtering effect, although decreases the photon number collected in the detection plane, enables the realization of a non-dispersive pseudo refractive index over the entire visible spectrum. Moreover, the pseudo refractive index can be flexibly designed to different values close to unity. Our angular-selective Brewster paradigm offers a feasible solution to implement compact and highly sensitive Cherenkov detectors especially in beam lines with a small angular divergence using regular dielectrics.

## Abstract (web summary)

Cherenkov detectors are used to detect high energy particles and their performance capabilities depend heavily on the material used. Here, the authors propose use of a Brewster-optics-based angular filter for a detector with increased sensitivity and particle identification capability.

## Introduction

A charged particle traveling in a transparent host medium emits photons when it travels faster than the phase velocity of the photons in that medium. This phenomenon is known as Cherenkov radiation, which was observed experimentally by P. A. Cherenkov (under the guidance of S. Vavilov)^1,2^ and later interpreted theoretically by I. M. Frank and I. Tamm^3,4^. Remarkably, Cherenkov radiation^5–8^ has enabled the invention of Cherenkov detectors^9–14^ for identifying particles over a large momentum range in high-energy physics and astrophysics. The Cherenkov detector has played an essential role in the discovery of many elementary particles, including anti-protons^15^, J/*ψ* particles^16,17^, neutrino oscillations^18^, etc.

According to Frank and Tamm’s theory of Cherenkov radiation^19^, the particle velocity *v* can be determined by measuring the light emission angle *θ*_CR_ (also known as the Cherenkov angle)

1 $${{{{{\rm{cos }}}}}}{\theta }_{{{{{{\rm{CR}}}}}}}=\frac{c}{{nv}},$$

where *n* is the refractive index of the host medium, and *c* is the speed of light in a vacuum. Although larger refractive indices give rise to lower Cherenkov thresholds and higher photon yield, they are not always desirable in Cherenkov detectors. The reason is that a large refractive index decreases the sensitivity of the Cherenkov angle to small changes in the velocity. In general, the highest detection sensitivity is obtained at *θ*_CR_→0 or *n→c/v* (detection sensitivity is defined as $\frac{d{\theta }_{{{{{{\rm{CR}}}}}}}}{dv}=\frac{n}{c}\cdot \frac{{\cos }^{2}{\theta }_{{{{{{\rm{CR}}}}}}}}{\sin \,{\theta }_{{{{{{\rm{CR}}}}}}}}$, which reaches its maximum at *θ*_CR_→0). In other words, to identify high-energy particles (i.e., *v*→*c*), transparent dielectrics with a near-unity refractive index are often required. Such a constraint limits the host materials used in many Cherenkov radiators to a low index. This is for example the case in ring imaging Cherenkov (RICH) detectors that use gases for the identification of particles with momenta larger than 10 GeV/*c*^14,20,21^.

Another limitation in state-of-the-art Cherenkov detectors is related to the photon emission efficiency: the use of low-index materials inevitably leads to low efficiency, since the photon yield is proportional to $1-\frac{{c}^{2}}{{n}^{2}{v}^{2}}\,$ according to Frank and Tamm’s theory. Especially when operating near-threshold (i.e., *v*→*c/n*), the photon yield approaches zero. Consequently, traditional gas radiators generally require bulky gas chambers to produce sufficient photons for detection^14^. Regular dielectrics offer a possible route to increase the photon emission efficiency, but their large refractive indices (generally far above unity) cause different types of high-energy particles to have nearly identical Cherenkov angles, namely *θ*_CR_→cos^−1^⁡(1/*n*), which then makes particle identification impossible. As an example, quartz has a refractive index around 1.4, and its corresponding momentum coverage for the identification of pions and kaons is currently limited to be below 6 GeV/*c*^22,23^. All high-energy particles with momenta above this value emit light at *θ*_CR_ ∈ [44.30°, 44.42°], independent of the particle velocity and particle type. Under this scenario, measuring *θ*_CR_ cannot lead to the identification of the corresponding particle type.

Recently, several theoretical attempts have been made to relax the material limitations in Cherenkov detectors by using modern concepts from nanophotonics and metamaterials^24,25^. One attempt proposes metal-based anisotropic metamaterials with one component of the effective refractive index close to unity^24^. Another study makes use of all-dielectric one-dimensional (1D) photonic crystals, where the constructive interference of resonance transition radiation is adopted to control the effective Cherenkov angle^25^. These nanophotonic Cherenkov detectors can achieve an enhanced sensitivity for any desired momentum range, however only at a specific working frequency. In fact, the working frequency is the major drawback of all nanophotonics-based Cherenkov detectors, i.e., they all have a narrow working bandwidth, resulting from the inherent chromatic dispersion of the constitutive materials (e.g., metal) and the resonant nature of periodic structures (e.g., photonic crystals). There are many more recent advances and ongoing efforts on the Cherenkov effect in nanophotonic settings and in emerging material platforms^26–37^. Nevertheless, the design of a broadband Cherenkov detector using regular transparent dielectrics and at the same time with enhanced performance has remained a long-standing scientific challenge.

**Fig. 1:**
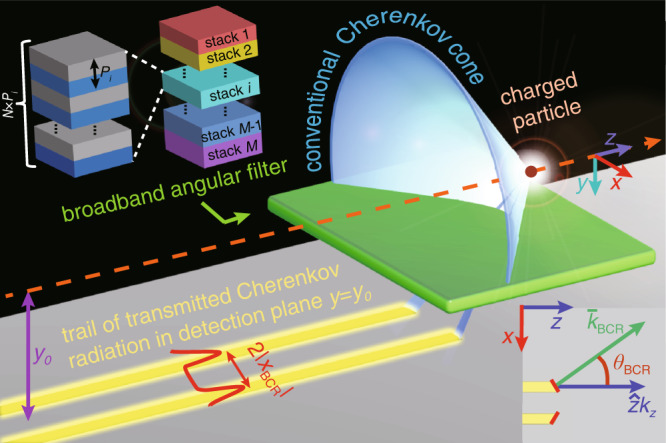
Schematic of the proposed Brewster-Cherenkov detector based on a broadband angular filter. In our design, the charged particle moves along a trajectory far away from the surface of the broadband angular filter. The broadband angular filter [see the structural schematic in the top-left inset] is designed by exploiting the Brewster effect in many stacks of 1D photonic crystals with various periodicities but two same constituent materials. After the angular filtering, the transmitted Cherenkov radiation in the detection plane has ${{{{{\rm{cos }}}}}}{\theta }_{{{{{{\rm{BCR}}}}}}}={k}_{z}/|{\bar{k}}_{{{{{{\rm{BCR}}}}}}}|$ [bottom-right inset], where *k*_*z*_ = *ω*/*v* and ${\bar{k}}_{{{{{{\rm{BCR}}}}}}}$ is the tangential wavevector of light parallel to the detection plane. The measurement of the peak-intensity position |*x*_BCR_/y_0_| of Cherenkov radiation in the detection plane provides the information of the pseudo Brewster-Cherenkov angle *θ*_BCR_ and the particle velocity, and it thus can be exploited for particle identification.

In this work, we propose a different paradigm for Cherenkov detectors by exploiting a broadband angular filter. This broadband angular filter is comprised of stacks of many 1D photonic crystals of different periodicities but identical constituent materials [Supplementary Fig. 1a]. As a result, the Brewster effect makes the angular filter transparent only to *p*-polarized (i.e., transverse magnetic, TM) light incident at the Brewster angle, while the light with other polarizations or incident at other angles is totally reflected. Moreover, we can readily tailor the band gaps of these 1D photonic crystals to cover a broad spectral range, thus also making the angular selectivity broadband, spanning the entire visible spectrum^38–41^. After transmission through the broadband angular filter, the Cherenkov radiation in the detection plane features a pseudo Brewster-Cherenkov angle *θ*_*BCR*_, namely the angle between the particle velocity and the tangential wavevector parallel to the detection plane [Fig. 1]. Remarkably, we find that the measurement of *θ*_*BCR*_ can provide an approach for particle identification at the desired momentum coverage with wide bandwidth and high sensitivity. This approach thus can tackle the key drawback listed above for nanophotonic Cherenkov detectors. Our approach is especially useful for the identification of a beam of charged particles with different momenta, even when the flux of charged particles is high.

## Results

### Brewster effect

We now proceed to analyze the essential role of the Brewster effect in our proposed particle detectors. As schematically shown in Fig. 1, we consider the charged particle traveling at a constant velocity *v* in a host material with a relative permittivity of *ε*_*h*_ and along a trajectory parallel to the surface of the broadband angular filter. Without loss of generality, we set the broadband angular filter composed of two regular transparent dielectrics with relative permittivities *ε*_r1_ and *ε*_r2_ [Supplementary Fig. 1a]. The detection plane is located at y = y_0_ beneath the broadband angular filter, and it is parallel to but far away from the particle trajectory [Fig. 1]. The Cherenkov radiation transmitting through the broadband angular filter has a tangential wavevector ${\bar{k}}_{{{{{{\rm{BCR}}}}}}}=\hat{x}{k}_{x}+\hat{z}{k}_{z}$ parallel to the detection plane. On the one hand, *k*_*z*_ = *ω*/*v* is fixed by the kinematic feature of the charged particle^25,42,43^. On the other hand, the magnitude of $\,{\bar{k}}_{{{{{{\rm{BCR}}}}}}}$ is locked by the intrinsic electromagnetic property of the broadband angular filter. To be specific, only the *p*-polarized light incident at the Brewster angle can transmit through our angular filter for the entire spectral range of 400 to 700 nm [Supplementary Figs. 1–3], and our particle detector will operate over this broadband wavelength range.

### Generalized Frank–Tamm formula

According to the Brewster effect, $|{\bar{k}}_{{{{{{\rm{BCR}}}}}}}|=\sqrt{\frac{{\varepsilon }_{{{{{{\rm{r}}}}}}1}{\varepsilon }_{{{{{{\rm{r}}}}}}2}}{{\varepsilon }_{{{{{{\rm{r}}}}}}1}+{\varepsilon }_{{{{{{\rm{r}}}}}}2}}}\frac{\omega }{c}$ ^20^, which enables us to define a pseudo refractive index

2 $${n}_{{{{{{\rm{BCR}}}}}}}=\sqrt{\frac{{\varepsilon }_{{{{{{\rm{r}}}}}}1}{\varepsilon }_{{{{{{\rm{r}}}}}}2}}{{\varepsilon }_{{{{{{\rm{r}}}}}}1}+{\varepsilon }_{{{{{{\rm{r}}}}}}2}}}.$$

We emphasize that the broadband angular filter is treated as a realistic multilayered structure, instead of an effectively homogeneous material, and hence *n*_BCR_ [see Methods for its derivations] is completely different from the effective refractive index obtained using the standard homogenization theory. Interestingly, *n*_BCR_ is related to the pseudo Brewster-Cherenkov angle *θ*_BCR_ [see inset of Fig. 1] by the following equation,

3 $${{{{{\rm{cos }}}}}}{\theta }_{{{{{{\rm{BCR}}}}}}}=\frac{{k}_{z}}{{{{{{\rm{|}}}}}}{\bar{k}}_{{{{{{\rm{BCR}}}}}}}{{{{{\rm{|}}}}}}}=\frac{c}{{n}_{{{{{{\rm{BCR}}}}}}}v}.$$

Equation (3) generalizes the regular Frank–Tamm formula (i.e., Eq. 1), providing a general route to engineer the Cherenkov radiation through the Brewster effect. In what follows, we shall explore the potential applications of this generalized Frank–Tamm formula for particle identification. As our approach exploits the Brewster effect, we refer to the particle detector depicted in Fig. 1 as the Brewster-Cherenkov (BCR) detector.

### Transmitted Cherenkov radiation in the detection plane

**Fig. 2:**
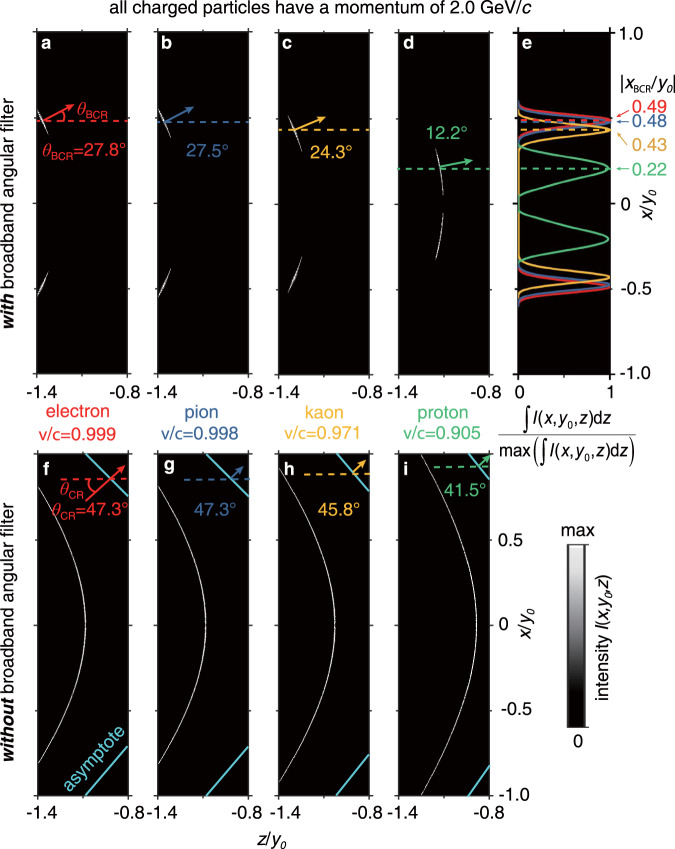
Cherenkov radiation in the detection plane of Brewster-Cherenkov detectors. Here we plot the intensity distribution of Cherenkov radiation for four elementary charged particles with a fixed momentum, when these charged particles arrive at *z* = 0. **a**–**e** The Brewster-Cherenkov detector has the broadband angular filter [Fig. 1]. **f**–**i** For comparison, the angular filter is removed. The Cherenkov radiation in (**a**–**d**) is related to the pseudo Brewster-Cherenkov angle *θ*_BCR_, whose value can be obtained through the measurement of the peak-intensity position in (**e**), while the asymptotic line of Cherenkov radiation in (**f**–**i**) is related to the regular Cherenkov angle *θ*_CR_. Our proposed approach has no critical requirement on the permittivity of the host material in which the charged particle is traveling (e.g., *ε*_h_ = *ε*_r1_ used in the calculation). Here and below, the considered wavelength varies from 400 to 700 nm. In addition, *y*_0_ = 2.3 mm, *ε*_r1_ = 2.18, and *n*_BCR_ = 1.13.

To facilitate the conceptual demonstration, Fig. 2 plots the spatial distribution of Cherenkov radiation in the detection plane. After penetrating the broadband angular filter, the transmitted Cherenkov photons feature two tails, which are symmetric with respect to the *z*-axis [Fig. 2a–d, Supplementary Fig. 4 and Supplementary Movie 1]. The number of Cherenkov photons in the detection plane of Brewster-Cherenkov detectors per unit length of the particle path is generally low but can be improved through the structural optimization of the broadband angular filter. Comparing the photon numbers to conventional RICH detectors, we find that both schemes can reach the same order of magnitude photon numbers [Supplementary Figs. 5–8]. For example, compared with the typical 20 photons per particle in RICH detectors with the same refractive index, the photon number in our detection plane can reach to 5 for electrons and pions, 7 for kaons, and 26 for protons, if all particle momenta are 2 GeV/*c* [Supplementary Figs. 5, 6]. The pseudo Brewster-Cherenkov angle, and hence the particle velocity, can be further determined with high sensitivity by measuring the peak-intensity position of Cherenkov radiation in the detection plane [Fig. 2e and Supplementary Fig. 9]. To illustrate the distinct advantages of our Brewster-Cherenkov detector, we also plot the Cherenkov radiation in the detection plane without the angular filter [Fig. 2f–i]. Through the comparison, we find that the pseudo Brewster-Cherenkov angle *θ*_BCR_ [Fig. 2a–d] is much more sensitive to the variation in particle velocity than the regular Cherenkov angle *θ*_CR_ [Fig. 2f–i]. This remarkable property clearly demonstrates that the broadband angular filter can effectively improve the sensitivity of Cherenkov detectors.

### Pseudo refractive index

**Fig. 3:**
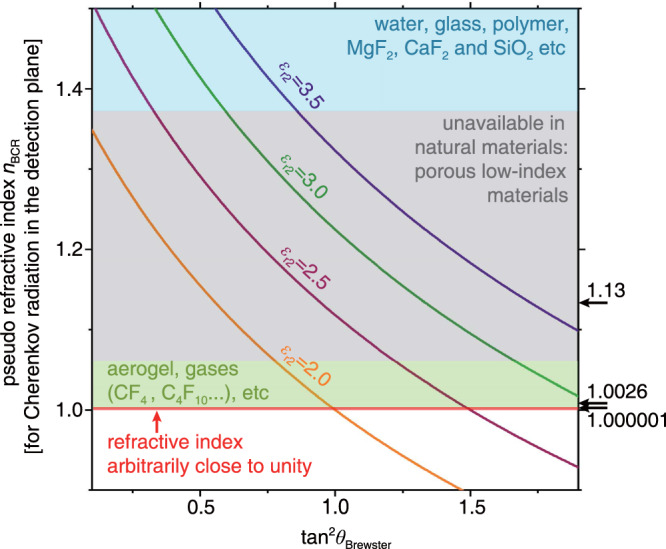
Engineering the pseudo refractive index *n*_BCR_ for Brewster-Cerenkov detectors, achieving parameter regimes that do not exist in natural materials. For the transmitted Cherenkov radiation in the detection plane, its wavevector component parallel to the detection plane has *k*_BCR_ = *n*_BCR_ *ω*/*c*, where ${n}_{{{{{{\rm{BCR}}}}}}}=\sqrt{\frac{{\varepsilon }_{{{{{{\rm{r}}}}}}1}{\varepsilon }_{{{{{{\rm{r}}}}}}2}}{{\varepsilon }_{{{{{{\rm{r}}}}}}1}+{\varepsilon }_{{{{{{\rm{r}}}}}}2}}}$. Here we plot *n*_BCR_ as a function of the Brewster angle *θ*_Brewster_ under different values of *ε*_r2_, where ${{{{{\rm{tan }}}}}}{\theta }_{{{{{{\rm{Brewster}}}}}}}=\sqrt{{\varepsilon }_{{{{{{\rm{r}}}}}}2}/{\varepsilon }_{{{{{{\rm{r}}}}}}1}}$.

We emphasize that such sensitivity improvement occurs over a broadband frequency range [Supplementary Fig. 10] because the pseudo refractive index *n*_BCR_ in the generalized Frank–Tamm formula can be made approximately nondispersive using regular transparent dielectrics that have a negligible dispersion in the visible range^44^. To illustrate this point, we plot *n*_BCR_ as a function of the Brewster angle. As shown in Fig. 3, *n*_BCR_ can be flexibly designed to values close to unity by a suitable choice of *ε*_r1_ and *ε*_r2_ for the two constituent materials of the broadband angular filter. For example, according to Eq. 2, *ε*_r1_ = 2.18 (e.g., SiO_2_) and *ε*_r2_ = 3.07 (Al_2_O_3_)^44^ give rise to a pseudo refractive index *n*_BCR_ = 1.13, which is much smaller (and hence more close to unity) than the lowest refractive index found in natural solid materials (i.e., 1.37 for MgF_2_)^20,21,44–47^. A pseudo refractive index even closer to unity can be achieved using other material combinations. For example, *n*_BCR_ = 1.0026, if taking polymers with *ε*_r1_ = 1.8578 (tetrafluoroethylene-co-hexafluoropropylene-co-vinylidene fluoride (THV, 3 M Dyneon 221AZ)) and *ε*_r2_ = 2.1904 (poly(methyl methacrylate), namely PMMA)^48–51^.

### Performance of Brewster-Cherenkov detectors

**Fig. 4:**
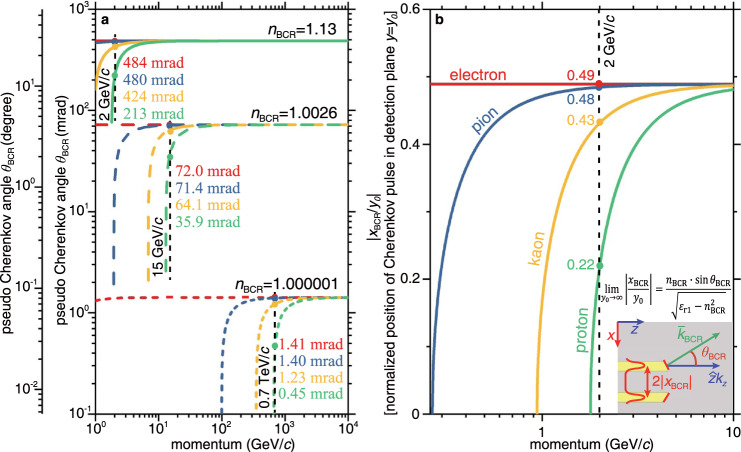
Performance of Brewster-Cherenkov detectors in the identification of high-energy particles. **a** Pseudo Brewster-Cherenkov angle *θ*_BCR_ versus particle momentum. This figure is plotted according to the generalized Frank–Tamm formula ${{{{{\rm{cos }}}}}}{\theta }_{{{{{{\rm{BCR}}}}}}}=\frac{c}{{n}_{{{{{{\rm{BCR}}}}}}}v}$, by transforming the particle velocity to the momentum. The values of pseudo Brewster-Cherenkov angles for four elementary particles, namely electron (red), pion (blue), kaon (yellow), and proton (green), with a momentum of 2, 15, or 700 GeV/*c* are given in the plot. **b** Normalized peak-intensity position |*x*_BCR_/*y*_0_| of the transmitted Cherenkov radiation in the detection plane versus particle momentum. The measurement of |*x*_BCR_/*y*_0_| provides the information of *θ*_BCR_ and further the particle velocity. In (b), *y*_0_ = 2.3 mm and *n*_BCR_ = 1.13.

A key feature of Brewster-Cherenkov detectors is that the pseudo refractive index *n*_BCR_ determines their sensitivity and momentum range. Fig. 4a shows the relation between the pseudo Brewster-Cherenkov angle *θ*_BCR_ and particle momenta for four elementary particles, namely electron, pion, kaon, and proton. In this exemplary case, we take *n*_BCR_ = 1.13 and a particle momentum fixed at 2 GeV/*c*. The resulting values of *θ*_BCR_ are 27.8° for electron, 27.5° for pion, 24.3° for kaon, and 12.2° for proton [Fig. 4a]. Such a variation in *θ*_BCR_ indicates that *n*_BCR_ = 1.13 is suitable for the identification of particles (such as pion (or electron), kaon, and proton) with a momentum less than 10 GeV/c. In comparison, *n*_BCR_ = 1.0026 gives rise to a Brewster-Cherenkov detector capable of identifying particles with a momentum larger than 10 GeV/*c*. More interestingly, when *n*_BCR_ = 1.000001, the corresponding Brewster-Cherenkov detector can even identify particles with ultra-high momenta in the TeV/*c* range. These results clearly demonstrate that the proposed Brewster-Cherenkov detectors can achieve high sensitivity within the desired momentum coverage through a proper choice of the pseudo refractive index. Covering a range of higher momenta requires further refinement of the detector design, for example using a larger number of layers for better angular resolution.

The pseudo Brewster-Cherenkov angle and the particle velocity can be determined by directly measuring the peak-intensity position *x*_BCR_ of Cherenkov radiation in the detection plane [Fig. 4b and Supplementary Figs. 11–13]. Mathematically, we have $\left|\frac{{x}_{{{{{{\rm{BCR}}}}}}}}{{y}_{0}}\right|=\frac{{n}_{{{{{{\rm{BCR}}}}}}}\cdot {{{{{\rm{sin }}}}}}{\theta }_{{{{{{\rm{BCR}}}}}}}}{\sqrt{{\varepsilon }_{{{{{{\rm{r}}}}}}1}-{n}_{{{{{{\rm{BCR}}}}}}}^{2}}}+\triangle ({y}_{0})$, where ∆(*y*_0_) is the normalized displacement resulting from the refraction of light through the broadband angular filter. For simplicity but without loss of generality, we choose *n*_BCR_ = 1.13 and *y*_0_ = 2.3 mm, and plot in Fig. 4b the ratio $\left|\frac{{x}_{{{{{{\rm{BCR}}}}}}}}{{y}_{0}}\right|$ as a function of momentum for the four elementary particles. At the fixed momentum of 2 GeV/*c*, the ratio $\left|\frac{{x}_{{{{{{\rm{BCR}}}}}}}}{{y}_{0}}\right|$ gradually varies from 0.49, 0.48, 0.43 to 0.22 for electrons, pions, kaons, and protons, respectively [e.g., see the corresponding intensity distributions of Cherenkov radiation in the detection plane in Fig. 2a–e].

## Discussion

As a final remark, we note that our Brewster approach has several unique advantages over the traditional methods for particle identification. First, the Brewster approach eliminates the strict requirement of near-unity-index host materials for the design of Cherenkov radiators. In other words, distinct from traditional Cherenkov detectors such as the RICH detector^13–16,18^, our Brewster approach does not have any special requirements on the refractive index of the host material where the particle is traveling, since the sensitivity of Brewster-Cherenkov detectors is directly determined by the *n*_BCR_ of the broadband angular filter. Consequently, high-index transparent solids or gases with low atomic numbers can now be used as the host material. Such a high-index host material can significantly enhance the number of photons penetrating the broadband angular filter and reaching the detection plane, enabling a higher-efficiency Cherenkov detector.

Another important advantage of our Brewster approach in comparison with all previous nanophotonic approaches^24,25^ is that the broadband angular filter does not need to be placed in the path of the high-energy particle beam. The charged particles can travel at a large distance away from the surface of the broadband angular filter so that all the Cherenkov photons are produced in the host material in which the particle is traveling. This way, the generation of secondary particles from the broadband angular filter can be effectively reduced. Last but not least, the performance of the proposed particle detector is robust to fabrication imperfections and geometric fluctuations of the broadband angular filter (see analysis in refs. ^38–41^). Therefore, this Brewster approach provides promising options to facilitate the design of advanced Cherenkov detectors with enhanced sensitivity, large bandwidth, miniaturized size, ultralightweight, and wide momentum coverage, all using readily available regular dielectrics. These enhanced capabilities are especially attractive in the identification of high-energy particles in beamlines with a small angular divergence. As illustrated in Supplementary Fig. 13, when the particle trajectory deviates from −0.5° to 0.5° with respect to the direction parallel to the top surface of the angular filter, the performance of Brewster-Cherenkov detectors remains almost unchanged.

## Methods

### Calculation of light emission from a charged particle moving parallel to the surface of the broadband angular filter

The basic structural setup is shown in Fig. 1. All the fields of emitted light are calculated in the framework of electromagnetic wave theory by applying the method of plane wave expansion. We let the *z*-axis parallel to the moving direction of a charged particle in Fig. 1; as such, the induced current density is $\bar{J}\left(\bar{r},t\right)=\hat{z}{qv}\delta \left(x\right)\delta \left(y\right)\delta (z-{vt})$. The field distribution in each region is obtained through matching the boundary conditions or by using the transfer matrix method. The detailed procedure of calculation is shown in Supplementary Note 1 of the supporting information.

### Derivation of the pseudo refractive index in Eq. 2 of the main text

For conceptual demonstration, here we set region 1 having the relative permittivity of *ε*_r1_ and region 2 having the relative permittivity of *ε*_r2_. By enforcing the electromagnetic boundary condition at the interface between regions 1 and 2, we can readily obtain the Brewster angle *θ*_Brewster_, at which the reflection for *p*-polarized waves is zero^19^. That is, according to the Brewster effect, the Brewster angle for *p*-polarized waves in region 1 has ${{{{{\rm{tan }}}}}}{\theta }_{{{{{{\rm{Brewster}}}}}}}=\sqrt{{\varepsilon }_{{{{{{\rm{r}}}}}}2}/{\varepsilon }_{{{{{{\rm{r}}}}}}1}\,}$^20^. At this Brewster angle, the wavevector component of light parallel to the interface has *k*_BCR_ = *k*_1_sin*θ*_Brewster_, where ${k}_{1}=\sqrt{{\varepsilon }_{{{{{{\rm{r}}}}}}1}}\omega /{c}$ is the wavevector of light in region 1 and ${\sin }{\theta }_{{{{{{\rm{Brewster}}}}}}}=\sqrt{{\varepsilon }_{{{{{{\rm{r}}}}}}2}}/\sqrt{{\varepsilon }_{{{{{{\rm{r}}}}}}1}+{\varepsilon }_{{{{{{\rm{r}}}}}}2}}$. In other words, we have ${k}_{{{{{{\rm{BCR}}}}}}}=\frac{\omega }{c}\sqrt{{\varepsilon }_{{{{{{\rm{r}}}}}}1}}\cdot \frac{\sqrt{{\varepsilon }_{{{{{{\rm{r}}}}}}2}}}{\sqrt{{\varepsilon }_{{{{{{\rm{r}}}}}}1}+{\varepsilon }_{{{{{{\rm{r}}}}}}2}}}=\frac{\omega }{c}\sqrt{\frac{{\varepsilon }_{{{{{{\rm{r}}}}}}1}{\varepsilon }_{{{{{{\rm{r}}}}}}2}}{{\varepsilon }_{{{{{{\rm{r}}}}}}1}+{\varepsilon }_{{{{{{\rm{r}}}}}}2}}}$. Since we denote ${k}_{{{{{{\rm{BCR}}}}}}}={n}_{{{{{{\rm{BCR}}}}}}}\frac{\omega }{c}$, we directly have Eq. 2 in the main text, namely ${n}_{{{{{{\rm{BCR}}}}}}}=\sqrt{\frac{{\varepsilon }_{{{{{{\rm{r}}}}}}1}{\varepsilon }_{{{{{{\rm{r}}}}}}2}}{{\varepsilon }_{{{{{{\rm{r}}}}}}1}+{\varepsilon }_{{{{{{\rm{r}}}}}}2}}}$. Due to the momentum matching at each interface, the value of *k*_BCR_ is the same for different regions in the broadband angular filter, when the light transmits through the angular filter. In addition, we highlight that all the calculations in this work treat the broadband angular filter as a realistic layered structure, instead of an effectively homogenized material by using the effective medium theory. These discussions are shown in Supplementary Note 2 of the supporting information.

### Design of the broadband angular filter

The broadband angular filter [Supplementary Fig. 1a] is comprised of many stacks (i.e., *M* stacks) of 1D photonic crystals. All 1D photonic crystals are made of two regular transparent dielectrics, which have a relative permittivity of *ε*_r1_ and ε_r2_, respectively. The *i*^th^ 1D photonic crystal has a pitch of _*Pi*_ = *d*_*i*1_ + *d*_*i*2_, where *d*_*i*1_ and *d*_*i*2_ are the thickness of two dielectric slabs in each pitch. All these 1D photonic crystals have a pitch number of *N* and a thickness ratio of *d*_*i*1_/*d*_*i*2_ = 3/2. This way, the band gap of each 1D photonic crystal can be flexibly tunable by changing _*Pi*_. The light transmission through the broadband angular filter is both angle-dependent and polarization-dependent. For the *p*-polarized light with arbitrary incident angle (except the one equal to the Brewster angle), the light is almost fully reflected by judiciously overlapping the band gaps of these 1D photonic crystals [Supplementary Figs. 1, 2]. For the *p*-polarized light incident at the Brewster angle, it can safely pass through the broadband angular filter with no reflection [Supplementary Figs. 1, 2]. For the *s*-polarized light, the transmission through the designed broadband angular filter is negligible for arbitrary incident angle [Supplementary Fig. 3]. The detailed design strategy is shown in Supplementary Notes 2–4 of the supporting information.

### Cherenkov radiation in the detection plane

For Cherenkov radiation passing through the broadband angular filter, while the direction of their in-plane wavevector ${\bar{k}}_{{{{{{\rm{BCR}}}}}}}=\hat{x}{k}_{x}+\hat{z}{k}_{z}$ has a pseudo Brewster-Cherenkov angle with respect to the particle trajectory [Supplementary Fig. 4], their motion in the detection plane is parallel to the particle trajectory (i.e., along the *z* axis); see the discussion of Cherenkov radiation in the detection plane in Supplementary Note 5 of the supporting information and their dynamics in the detection plane in Supplementary Movie 1. Moreover, the number of Cherenkov photons in the detection plane [Supplementary Figs. 5, 6] and the corresponding width of their intensity distribution [Supplementary Figs. 7, 8] are two key parameters for Cherenkov detectors. The dependences of these two parameters on the geometric property of the broadband angular filter are also discussed in Supplementary Note 5.

### Peak-intensity position of Cherenkov radiation in the detection plane at y = y_0_

The normalized peak-intensity position $|\frac{{x}_{{{{{{\rm{BCR}}}}}}}}{{y}_{0}}|=\frac{{n}_{{{{{{\rm{BCR}}}}}}}\cdot {{{{{\rm{sin }}}}}}{\theta }_{{{{{{\rm{BCR}}}}}}}}{\sqrt{{\varepsilon }_{{{{{{\rm{r}}}}}}1}-{n}_{{{{{{\rm{BCR}}}}}}}^{2}}}+\triangle ({y}_{0})$ is analytically calculated according to the ray-tracing theory. Here, $\triangle \left({y}_{0}\right)=-\left(\frac{\sqrt{{n}_{{{{{{\rm{BCR}}}}}}}^{2}-\frac{{c}^{2}}{{v}^{2}}}}{\sqrt{{\varepsilon }_{{{{{{\rm{r}}}}}}1}-{n}_{{{{{{\rm{BCR}}}}}}}^{2}}}-\frac{\sqrt{{n}_{{{{{{\rm{BCR}}}}}}}^{2}-\frac{{c}^{2}}{{v}^{2}}}}{\sqrt{{\varepsilon }_{{{{{{\rm{r}}}}}}2}-{n}_{{{{{{\rm{BCR}}}}}}}^{2}}}\right)\frac{{y}_{b}}{{y}_{0}}$ is the normalized displacement induced by the refraction of light through the broadband angular filter, where *y*_*b*_ is the total thickness of dielectric regions with *ε*_r2_ in the designed broadband angular filter. Since $\triangle \left({y}_{0}\right)\propto \frac{{y}_{b}}{{y}_{0}}$, we have ∆(*y*_0_) → 0 if *y*_*b*_ ≪ *y*_0_. In other words, if the detection plane is far away from the particle trajectory and if their distance is much larger than the finite thickness of the broadband angular filter, we have $\left|\frac{{x}_{{{{{{\rm{BCR}}}}}}}}{{y}_{0}}\right|=\frac{{n}_{{{{{{\rm{BCR}}}}}}}\cdot {{\sin }}{\theta }_{{{{{{\rm{BCR}}}}}}}}{\sqrt{{\varepsilon }_{{{{{{\rm{r}}}}}}1}-{n}_{{{{{{\rm{BCR}}}}}}}^{2}}}$. The detailed discussion of $\left|\frac{{x}_{{{{{{\rm{BCR}}}}}}}}{{y}_{0}}\right|$ for a fixed broadband angular filter under different values of *y*_0_ is shown in Supplementary Note 6 and Supplementary Figs. 9–12 in the supporting information.

### Robustness of the performance of Brewster-Cherenkov detectors to particle’s trajectory

The Brewster-Cherenkov detector has the potential to infer the projection of particle trajectory in the *xz* plane (or the detection plane at *y* = *y*_0_). This is because the intensity distribution of the transmitted Cherenkov radiation in the detection plane is symmetric with respect to the projection of the particle trajectory in the *xz* plane [Fig. 2a–e]. Due to this unique feature, the sensitivity of Brewster-Cherenkov detectors is in principle insensitive to the direction of particle velocity, if the particle velocity is parallel to the surface of the broadband angular filter. On the other hand, for the Brewster-Cherenkov detector, we can always set the particle trajectory very far away from the top surface of the broadband angular filter. Then if the particle velocity is unparallel to the surface of the broadband angular filter and tilted by a small angle *α* (e.g., |*α*|≤0.5° [Supplementary Fig. 13]) (but the particle would not penetrate through the filter), the performance of Brewster-Cherenkov detectors would not be degraded, since the feature of the transmitted Cherenkov radiation in the detection plane is mostly preserved.

### Influence of the finite thickness of the broadband angular filter on the performance of Brewster-Cherenkov detectors

More discussions on the performance of Brewster-Cherenkov detectors are provided in Supplementary Note 7 and Supplementary Figs. 14–16 of the supporting information. When the stack number *M* of 1D photonic crystals and the periodicity number *N* of each 1D photonic crystal are finite, the *p*-polarized light incident at the angles very close to the Brewster angle can also safely pass through the broadband angular filter. This way, there is a small angular (and thus spatial) spread of the transmitted Cherenkov radiation in the detection plane, such as those shown in Supplementary Fig. 1b–e. This phenomenon would degrade the sensitivity of Brewster-Cherenkov detectors. However, the sensitivity of the Brewster-Cherenkov detector can still be guaranteed by effectively avoiding this phenomenon, through increasing both the values of *M* and *N* in the practical implementation [Supplementary Fig. 15].

## Supplementary information

Supplementary Information

Description of Additional Supplementary Files

Supplementary Movie 1

## Data Availability

All relevant data are available from the authors upon reasonable request.
