# Supplementary material for: A Brewster route to Cherenkov detectors
Source: Nat Commun. 2021 Sep 21;12:5554. doi: 10.1038/s41467-021-25822-x (PMC8455627; doi:10.1038/s41467-021-25822-x)
Supplement: Supplementary file 1 — Supplementary Information [file 41467_2021_25822_MOESM1_ESM.pdf]

Supplementary Information for

“A Brewster route to Cherenkov detectors”

Xiao Lin, Hao Hu, Sajan Easo, Yi Yang, Yichen Shen, Kezhen Yin, Michele Piero Blago, Ido Kaminer, Baile Zhang, Hongsheng Chen, John Joannopoulos, Marin Soljačić, and Yu Luo

Supplementary Information Guide:

-- Supplementary Methods

--Supplementary Note 1. Calculation of light emission from a charged particle moving parallel to the surface of the broadband angular filter.

-- Supplementary Note 2. Broadband angular filter for Brewster-Cherenkov detectors and more discussion on Fig. 2a-d

-- Supplementary Note 3. Electromagnetic property of the broadband angular filter for *p*-polarized light.

-- Supplementary Note 4. Electromagnetic property of the broadband angular filter for *s*-polarized light.

-- Supplementary Note 5. Field distribution and photon number of Cherenkov radiation in the detection plane.

-- Supplementary Note 6. Peak-intensity position of Cherenkov radiation in the detection plane.

-- Supplementary Note 7. More discussions on the performance of Brewster-Cherenkov detectors.

Supplementary Methods

<u>Supplementary Note 1: Calculation of light emission from a charged particle moving parallel to the surface of the broadband angular filter.</u>

We begin with the analytical calculation of Cherenkov radiation created from a charged particle moving with its trajectory parallel to the top surface of the broadband angular filter [Fig. 1]. The broadband angular filter is comprised of many stacks of 1D photonic crystals [Supplementary Fig. 1a]. In our design, the proposed particle detector can perform well when the charged particle is very far away from the surface of the broadband angular filter. Such a particular setup effectively avoids the direct interaction between the charged particle and the broadband angular filter. Therefore, the proposed structure in Fig. 1 can maintain a low rate of secondary particle production triggered by the original particle.

The induced current density by the charged particle with a velocity of $\bar{v} = \hat{z}v$ is

$$\bar{J}(\bar{r}, t) = \hat{z}vq\delta(x)\delta(y)\delta(z - vt) \tag{1.1}$$

By applying the Fourier transformation, the current density in the frequency domain is

$$\bar{J}(\bar{r}, \omega) = \frac{1}{2\pi}\int \mathrm{d}t\,\bar{J}(\bar{r}, t)e^{\mathrm{i}\omega t} = \hat{z}\frac{q}{4\pi^2\rho}e^{\mathrm{i}\frac{\omega}{v}z}\delta(\rho) \tag{1.2}$$

where q is the elementary charge. Correspondingly, the induced electric and magnetic fields in the cylindrical coordinates [19] can be expressed as

$$\bar{E}(\bar{r}, \omega) = \frac{-q}{8\pi\omega\varepsilon_0\varepsilon_\mathrm{b}}\left(\hat{z}k_\rho^2 + \mathrm{i}\frac{\omega}{v}\nabla\right)\mathrm{H}_0^{(1)}\left(k_\rho\rho\right)e^{\mathrm{i}\frac{\omega}{v}z} \tag{1.3}$$

$$\bar{H}(\bar{r}, \omega) = \hat{\varphi}\frac{\mathrm{i}qk_\rho}{8\pi}H_1^{(1)}(k_\rho\rho)e^{\mathrm{i}\frac{\omega}{v}z} \tag{1.4}$$

where $k_\rho = \sqrt{\varepsilon_\mathrm{h}k_0^2 - k_z^2}$, $k_0 = \omega/c$, and ε_h is the relative permittivity of the host material in which the charged particle moves. In the main text, we set that the dielectric regions above and below the broadband angular filter both have ε_h. For simplicity, we let $\varepsilon_\mathrm{h} = \varepsilon_\mathrm{r1}$ in the numerical calculation in this work.

To facilitate the analytical calculation, a vector potential $\bar{A} = \hat{z}\phi_{\mathrm{TM},0}$ is introduced below so that the equations (1.3-1.4) are equivalent to [19]

$$\bar{E}(\bar{r},\omega) = \frac{\mathrm{i}}{\omega\varepsilon_0\varepsilon_{\mathrm{h}}}\nabla\times\nabla\times\bar{A} \tag{1.5}$$

$$\bar{H}(\bar{r},\omega) = \nabla\times\bar{A} \tag{1.6}$$

After some calculation, the scalar potential $\phi_{\mathrm{TM},0}$ is obtained as

$$\phi_{\mathrm{TM},0} = \frac{\mathrm{i}q}{8\pi}\mathrm{H}_0^{(1)}(k_\rho\rho)\mathrm{e}^{\mathrm{i}k_z z} = \int_{-\infty}^{+\infty}\mathrm{d}k_x\frac{\mathrm{i}q}{8\pi^2 k_y}\mathrm{e}^{\mathrm{i}k_x x+\mathrm{i}k_y|y|+\mathrm{i}k_z z} \tag{1.7}$$

where $k_z = \frac{\omega}{v}$ and $k_y = \sqrt{\varepsilon_{\mathrm{h}}k_0^2 - k_z^2 - k_x^2}$ are the components of wavevector along the z and y directions, respectively. For Cherenkov radiation, it can propagate to the far field only if k_y is a real number. As such, the regular Cherenkov radiation in the host material arises only if $v > v_{\mathrm{th}}$, where $v_{\mathrm{th}} = \frac{c}{\sqrt{\varepsilon_{\mathrm{h}}}}$ is known as the Cherenkov threshold in the host material.

For the structural and coordinate setup in Fig. 1, only the *p*-polarized light has the field component of E_y, and only the *s*-polarized light has the field component of H_y. The broadband angular filter in Fig. 1 is judiciously designed to be transparent only to the *p*-polarized light with the incident angle equal to the Brewster angle. This way, below we first focus on the analytical calculation of the emitted *p*-polarized light from the charged particle. That is, we first calculate E_y in the regions below the particle trajectory, and then go to the calculation of H_y in the regions below the particle trajectory by following a similar procedure.

From the scalar potential $\phi_{\mathrm{TM},0}$, E_y can be written as [36]

$$E_y = \frac{\mathrm{i}}{\omega\varepsilon}\frac{\partial^2\phi_{\mathrm{TM},0}}{\partial y\partial z} = -\frac{\mathrm{i}}{\omega\varepsilon_0}\int_{-\infty}^{+\infty}\mathrm{d}k_x\mathrm{sgn}(y)\left(\frac{\mathrm{i}q}{8\pi^2 k_y}\right)\left(\frac{k_y k_z}{\varepsilon_{\mathrm{h}}}\right)\mathrm{e}^{\mathrm{i}k_x x+\mathrm{i}k_y|y|+\mathrm{i}k_z z} \tag{1.8}$$

Recall that the broadband angular filter in Fig. 1 is a 1D layered structure and is beneath the particle trajectory. Then the scalar potential $\phi_{\mathrm{TM},j}$ in the jth region takes the following form

$$\phi_{\mathrm{TM},j} = \int_{-\infty}^{+\infty} \mathrm{d}k_x \left(T_{p,j}^{+} e^{\mathrm{i}k_{y,j}(y-y_j)} + R_{p,j}^{+} e^{-\mathrm{i}k_{y,j}(y-y_j)}\right) \mathrm{e}^{\mathrm{i}k_x x + \mathrm{i}k_z z} \tag{1.9}$$

where y_j is the position of boundary between the $(j-1)^{\mathrm{th}}$ and j^{th} regions. To facilitate the calculation, the host material in which the particle moves is denoted as the 0th region. Accordingly, the field E_y in the jth region is

$$E_{y,j} = \frac{\mathrm{i}}{\omega\varepsilon_0} \int_{-\infty}^{+\infty} \mathrm{d}k_x \left(-\frac{k_{y,j}k_z}{\varepsilon_{\mathrm{r},j}} T_{p,j}^{+} \mathrm{e}^{\mathrm{i}k_{y,j}(y-y_j)} + \frac{k_{y,j}k_z}{\varepsilon_{\mathrm{r},j}} R_{p,j}^{+} \mathrm{e}^{-\mathrm{i}k_{y,j}(y-y_j)}\right) \mathrm{e}^{\mathrm{i}k_x x + \mathrm{i}k_z z} \tag{1.10}$$

Below we denote $E_{\parallel,j}$ as the component of electric field parallel to the incidence plane of light with a certain k_x, and we denote $H_{\perp,j}$ as the component of magnetic field vertical to the incidence plane with k_x. Both $E_{\parallel,j}$ and $H_{\perp,j}$ are parallel to the surface of the broadband angular filter. By applying the Gauss's law ($\nabla \cdot \bar{E} = 0$) and the Faraday's law ($\nabla \times \bar{E} = \mathrm{i}\omega\mu_0\bar{H}$), these field components $E_{\parallel,j}$ and $H_{\perp,j}$ of the p-polarized light can be calculated as

$$E_{\parallel,j} = \frac{\mathrm{i}}{\omega\varepsilon_0} \left(\frac{{k_{y,j}}^2 k_z}{\varepsilon_{\mathrm{r},j} k_{\parallel,j}} T_{p,j}^{+} \mathrm{e}^{\mathrm{i}k_{y,j}(y-y_j)} + \frac{{k_{y,j}}^2 k_z}{\varepsilon_{\mathrm{r},j} k_{\parallel,j}} R_{p,j}^{+} \mathrm{e}^{-\mathrm{i}k_{y,j}(y-y_j)}\right) \mathrm{e}^{\mathrm{i}k_x x + \mathrm{i}k_z z} \tag{1.11}$$

$$H_{\perp,j} = \mathrm{i} \left(\frac{k_{y,j}k_z}{k_{\parallel,j}} T_{p,j}^{+} \mathrm{e}^{\mathrm{i}k_{y,j}(y-y_j)} - \frac{k_{y,j}k_z}{k_{\parallel,j}} R_{p,j}^{+} \mathrm{e}^{-\mathrm{i}k_{y,j}(y-y_j)}\right) \mathrm{e}^{\mathrm{i}k_x x + \mathrm{i}k_z z} \tag{1.12}$$

where the in-plane wavevector of these fields has $\bar{k}_{\parallel,j} = k_x\hat{x} + k_z\hat{z}$. By matching the boundary conditions (i.e., the continuity of $E_{\parallel,j}$ and $H_{\perp,j}$ at each boundary), we have

$$\frac{{k_{y,j-1}}^2}{\varepsilon_{\mathrm{r},j-1} k_{\parallel,j-1}} T_{p,j-1}^{-} + \frac{{k_{y,j-1}}^2}{\varepsilon_{\mathrm{r},j-1} k_{\parallel,j-1}} R_{p,j-1}^{-} = \frac{{k_{y,j}}^2}{\varepsilon_{\mathrm{r},j} k_{\parallel,j}} T_{p,j}^{+} + \frac{{k_{y,j}}^2}{\varepsilon_{\mathrm{r},j} k_{\parallel,j}} R_{p,j}^{+} \tag{1.13}$$

$$\frac{k_{y,j-1}}{k_{\parallel,j-1}} T_{p,j-1}^{-} - \frac{k_{y,j-1}}{k_{\parallel,j-1}} R_{p,j-1}^{-} = \frac{k_{y,j}}{k_{\parallel,j}} T_{p,j}^{+} - \frac{k_{y,j}}{k_{\parallel,j}} R_{p,j}^{+} \tag{1.14}$$

The above equations can be sorted into a compact matrix form, that is,

$$\begin{bmatrix} R_{p,j-1}^{-} \\ T_{p,j}^{+} \end{bmatrix} = S_{1j} \begin{bmatrix} T_{p,j-1}^{-} \\ R_{p,j}^{+} \end{bmatrix}, \tag{1.15}$$

where the scattering matrix S_{1j} at the boundary of $y = y_j$ is

$$S_{1j} = \frac{1}{\frac{k_{y,j-1}}{\varepsilon_{\mathrm{r},j-1}} + \frac{k_{y,j}}{\varepsilon_{\mathrm{r},j}}} \begin{bmatrix} \frac{k_{y,j}}{\varepsilon_{\mathrm{r},j}} - \frac{k_{y,j-1}}{\varepsilon_{\mathrm{r},j-1}} & 2\frac{k_{y,j}}{\varepsilon_{\mathrm{r},j}} \frac{k_{\parallel,j-1} k_{y,j}}{k_{\parallel,j} k_{y,j-1}} \\ 2\frac{k_{y,j-1}}{\varepsilon_{\mathrm{r},j-1}} \frac{k_{\parallel,j} k_{y,j-1}}{k_{\parallel,j-1} k_{y,j}} & \frac{k_{y,j-1}}{\varepsilon_{\mathrm{r},j-1}} - \frac{k_{y,j}}{\varepsilon_{\mathrm{r},j}} \end{bmatrix}.$$

For the jth region with a thickness of $h_j = y_{j+1} - y_j$, the reflection and transmission coefficients are determined by

$$\begin{bmatrix} R_{p,j}^{+} \\ T_{p,j}^{-} \end{bmatrix} = S_{2j} \begin{bmatrix} T_{p,j}^{+} \\ R_{p,j}^{-} \end{bmatrix}, \qquad (1.16)$$

where the scattering matrix S_{2j} in the jth region is

$$S_{2j} = \begin{bmatrix} 0 & e^{\mathrm{i}k_{y,j}h_j} \\ e^{\mathrm{i}k_{y,j}h_j} & 0 \end{bmatrix}.$$

Given the incidence amplitude of light being $T_{p,0}^{+} = \frac{\mathrm{i}q}{8\pi^2 k_y}$ and the reflection from the infinity being $R_{p,g}^{-} = 0$, all the coefficients ($T_{p,1}^{\pm}, T_{p,2}^{\pm}, \dots, T_{p,g}^{\pm}$ and $R_{p,1}^{\pm}, R_{p,2}^{\pm}, \dots, R_{p,g}^{\pm}$) can be solved with the method of transfer matrix [25,37]. Here $y = y_g$ is the last boundary of the configuration. With the knowledge of E_y in the frequency domain, the time-domain distribution of the electric field $E_y(x, y, z, t)$ at $y = y_0$ can be expressed as

$$E_y(x, y_0, z, t) = 2\mathrm{Re}\{\int_0^{+\infty} \mathrm{d}\omega\, E_y(x, y_0, z, \omega) e^{-\mathrm{i}\omega t}\} \qquad (1.19)$$

Accordingly, for the calculation of the *s*-polarized light emitted from the charged particle, one can express the field component H_y as

$$H_y = -\frac{\partial \phi_{\mathrm{TM},0}}{\partial x} = \mathrm{i}\int_{-\infty}^{+\infty} (-k_x) \mathrm{d}k_x \left(\frac{\mathrm{i}q}{8\pi^2 k_y}\right) \mathrm{e}^{\mathrm{i}k_x x + \mathrm{i}k_y |y| + \mathrm{i}k_z z} \qquad (1.20)$$

After a similar calculation procedure to that of the *p*-polarized emitted light, the matrix equation at the boundary of $y = y_j$ for the *s*-polarized emitted light is obtained as

$$\begin{bmatrix} R_{s,j-1}^{-} \\ T_{s,j}^{+} \end{bmatrix} = S_{3j} \begin{bmatrix} T_{s,j-1}^{-} \\ R_{s,j}^{+} \end{bmatrix} \tag{1.21}$$

The scattering matrix in equation (1.21) is $\mathrm{S}_{3j} = \frac{1}{k_{y,j-1}+k_{y,j}} \begin{bmatrix} k_{y,j-1} - k_{y,j} & 2k_{y,j}\frac{k_{\parallel,j-1}}{k_{\parallel,j}} \\ 2k_{y,j-1}\frac{k_{\parallel,j}}{k_{\parallel,j-1}} & k_{y,j} - k_{y,j-1} \end{bmatrix}$.

Inside the jth region of the broadband angular filter, the scattering matrix equation is

$$\begin{bmatrix} R_{s,j}^{+} \\ T_{s,j}^{-} \end{bmatrix} = \mathrm{S}_{4j} \begin{bmatrix} T_{s,j}^{+} \\ R_{s,j}^{-} \end{bmatrix} \tag{1.22}$$

The scattering matrix in equation (1.22) is $\mathrm{S}_{4j} = \begin{bmatrix} 0 & e^{\mathrm{i}k_{y,j}h_j} \\ e^{\mathrm{i}k_{y,j}h_j} & 0 \end{bmatrix}$.

Similarly, the transmission and reflection coefficients for the *s*-polarized light ($T_{s,1}^{\pm}, T_{s,2}^{\pm}, \dots, T_{s,g}^{\pm}$ and $R_{s,1}^{\pm}, R_{s,2}^{\pm}, \dots, R_{s,g}^{\pm}$) can also be solved by the method of transfer matrix.

Supplementary Note 2. Broadband angular filter for Brewster-Cherenkov detectors and more discussion on Fig. 2a-d

This section serves as the complementary information for Figs. 1&2a-d. Supplementary Fig. 1a shows the structural schematic of the broadband angular filter, which is used for the Brewster-Cherenkov detector in Fig. 1. When the light emitted from a swift charged particle interacts with the broadband angular filter, only the *p*-polarized light incident at the Brewster angle can safely pass through the broadband angular filter. This information can be concluded from Supplementary Fig. 1b-e, which shows the distribution of electric field E_y for the transmitted Cherenkov radiation in the detection plane as a function of working wavelength and the pseudo Brewster-Cherenkov angle. All structural setup in Supplementary Fig. 1b-e are the same as Fig. 2a-d.

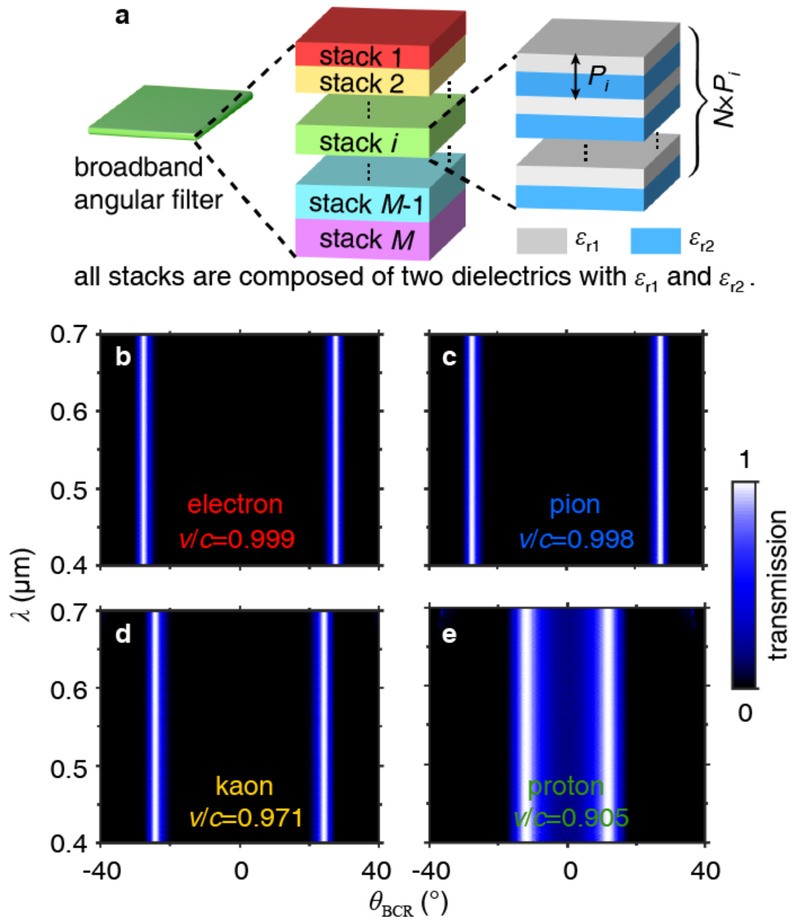

Supplementary Figure 1 | Broadband angular filter for the Brewster-Cherenkov detector. a, Schematic of the broadband angular filter, comprised of two transparent dielectrics. **b-e**, Transmission coefficient for the p-polarized light as a function of the wavelength and the pseudo Brewster-Cherenkov angle θ_{BCR}. For the transmitted Cherenkov radiation, $\cos\theta_{\mathrm{BCR}} = k_z/k_{\mathrm{BCR}}$, where $k_z = \omega/v$ and $\bar{k}_{\mathrm{BCR}}$ is the tangential wavevector of incident light parallel to the detection plane. Four elementary particles with a fixed momentum of 2 GeV/c are considered in (b-e). By judiciously overlapping the band gaps of different 1D photonic crystals, the broadband angular filter only allows the p-polarized light incident at the Brewster angle to pass through. A thicker broadband angular filter has a better performance in the angular filtering [Supplementary Fig. 15]. The results in (b-e) serves as the complementary information for Fig. 2a-d; all structural setup in (b-e) are the same as Fig. 2a-d.

Derivation of the pseudo refractive index in equation (2) of the main text.

For conceptual demonstration, here we set region 1 having the relative permittivity of $\varepsilon_{\mathrm{r1}}$ and region 2 having the relative permittivity of $\varepsilon_{\mathrm{r2}}$. By enforcing the electromagnetic boundary condition at the interface between regions 1 & 2, we can readily obtain the Brewster angle $\theta_{\mathrm{Brewster}}$, at which the reflection for *p*-polarized waves is zero [19]. That is, according to the Brewster effect, the Brewster angle for *p*-polarized waves in region 1 has $\tan\theta_{\mathrm{Brewster}} = \sqrt{\varepsilon_{\mathrm{r2}}/\varepsilon_{\mathrm{r1}}}$ [19]. At this Brewster angle, the wavevector component of light parallel to the interface has $k_{\mathrm{BCR}} = k_1 sin\theta_{\mathrm{Brewster}}$, where $k_1 = \sqrt{\varepsilon_{\mathrm{r1}}}\omega/c$ is the wavevector of light in region 1. In other words, we have $k_{\mathrm{BCR}} = \frac{\omega}{c}\sqrt{\varepsilon_{\mathrm{r1}}}\cdot\frac{\sqrt{\varepsilon_{\mathrm{r2}}}}{\sqrt{\varepsilon_{\mathrm{r1}}+\varepsilon_{\mathrm{r2}}}} = \frac{\omega}{c}\sqrt{\frac{\varepsilon_{\mathrm{r1}}\varepsilon_{\mathrm{r2}}}{\varepsilon_{\mathrm{r1}}+\varepsilon_{\mathrm{r2}}}}$. Since we denote $k_{\mathrm{BCR}} = n_{\mathrm{BCR}}\frac{\omega}{c}$, we directly have equation (2) in the main text, namely $n_{\mathrm{BCR}} = \sqrt{\frac{\varepsilon_{\mathrm{r1}}\varepsilon_{\mathrm{r2}}}{\varepsilon_{\mathrm{r1}}+\varepsilon_{\mathrm{r2}}}}$. Due to the momentum matching at each interface, the value of k_{BCR} is the same for different regions in the broadband angular filter, when the light transmits through the broadband angular filter. In addition, we highlight that all calculations in this work treat the broadband angular filter as a realistic layered structure, instead of an effectively homogenized material by using the effective medium theory.

Supplementary Note 3: Electromagnetic property of the broadband angular filter for *p*-polarized light

In this section, we briefly introduce the design strategy for the broadband angular filter, which is featured by the broadband angular selectivity phenomenon for the *p*-polarized light. The Brewster effect shows that the *p*-polarized light at any dielectric boundary is reflection-free if the incident angle is equal to the Brewster angle. As such, the *p*-polarized light can safely pass through the 1D photonic crystal comprised of two regular transparent dielectrics, if the incident angle is equal to the Brewster angle. At the Brewster angle, the *p*-polarized light has an in-plane (i.e., parallel to the boundary) wavevector $|\bar{k}_{\parallel}| = k_{\mathrm{BCR}}$, where $k_{\mathrm{BCR}} = \sqrt{\frac{\varepsilon_{\mathrm{r1}}\varepsilon_{\mathrm{r2}}}{\varepsilon_{\mathrm{r1}}+\varepsilon_{\mathrm{r2}}}}k_0 = n_{\mathrm{BCR}}k_0$ according to the Brewster effect. When the incident angle is not equal to the

Brewster angle, the *p*-polarized light will be fully reflected if the frequency is within the photonic band gap of one 1D photonic crystal. The wavelength range for the photonic band gap is dependent on the structural periodicity [Supplementary Fig. 2]. If many 1D photonic crystals with different periodicities but the same constituent materials (i.e., two regular transparent dielectrics) are stacked together, the broadband angular filter can be flexibly constructed; see one example in Supplementary Fig. 2.

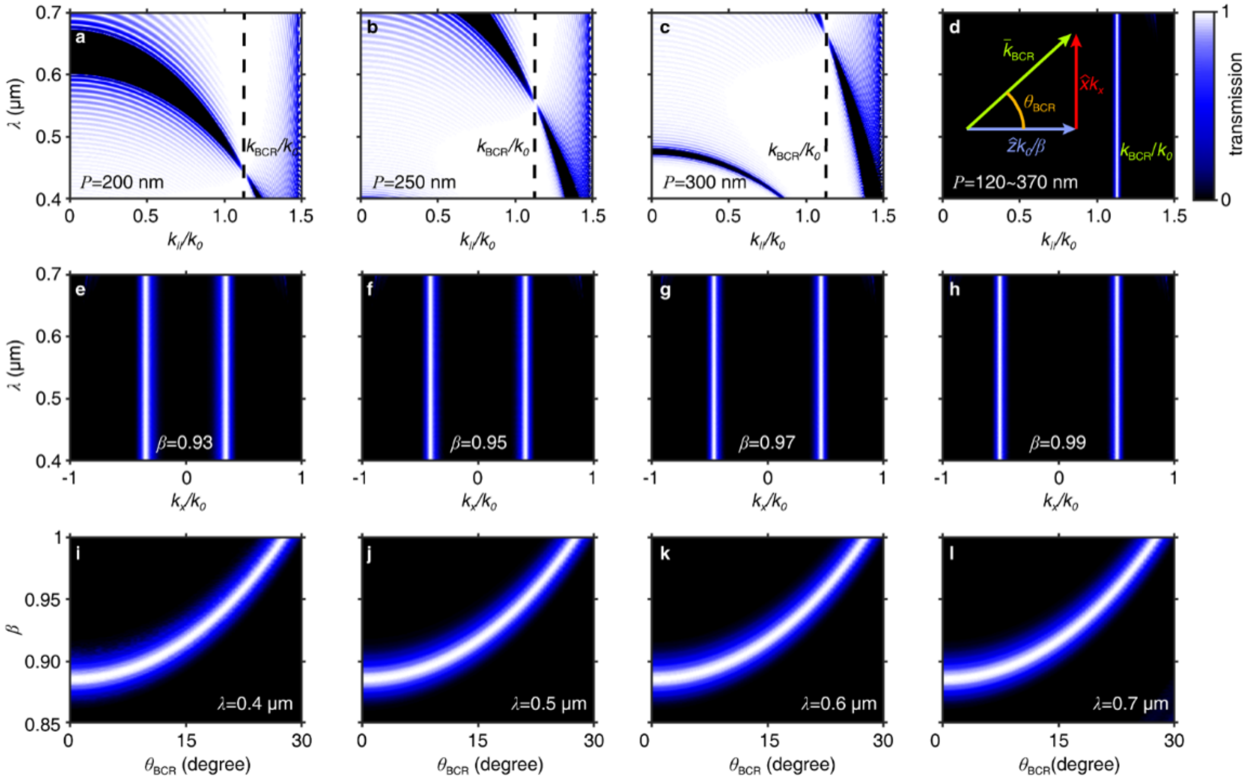

Supplementary Figure 2 | Electromagnetic property of the designed broadband angular filter for *p*-polarized light. As schematically shown in Supplementary Fig. 1a, the broadband angular filter is comprised of many stacks (i.e., M stacks) of individual 1D photonic crystals. These 1D photonic crystals are made of two regular transparent dielectrics, which have a relative permittivity of ε_{r1} and ε_{r2}, respectively. The ith 1D photonic crystal has a pitch of $P_i = d_{i1} + d_{i2}$, where d_{i1} and d_{i2} are the thickness of two dielectric slabs in each pitch. For all 1D photonic crystals in this work, we choose $d_{i1}/d_{i2} = 3/2$ and set the periodicity number to be $N = 50$. This way, the band gap of each 1D photonic crystal can be flexibly tunable by changing the value of P_i. **a-c**, Transmission of the *p*-polarized light through a 1D

photonic crystal. The periodicity is 200 nm in (a), 250 nm in (b) and 300 nm in (c). **d**, Transmission of the p-polarized light through the designed broadband angular filter. Here, $M = 200$, $P_0 = 120$ nm, $P_i = P_0 \times \left(\sqrt[M-1]{P_M/P_0}\right)^{i-1}$, and $P_M = 370$ nm. By judiciously overlapping the band gaps of these 1D photonic crystals, the light transmission for the p-polarized light with arbitrary incident angle (except the one equal to the Brewster angle) is almost zero. In contrast, for the p-polarized light incident with the Brewster angle, the transmission is unity. **e-h**, Transmission as a function of the working wavelength in free space and the value of k_x under four different values of β. The value $\beta = v/c$ represents the kinematic feature of the charged particle and determines the value of k_z. To be specific, $k_z = \omega/v$. The value of β is 0.93 in (e), 0.95 in (f), 0.97 in (g), and 0.99 in (h). **i-l**, Transmission for the p-polarized light as a function of β and the pseudo Brewster-Cherenkov angle θ_{BCR} at different working wavelengths. For Cherenkov radiation passing through the broadband angular filter, the pseudo Brewster-Cherenkov angle is the angle between the component of wavevector parallel to the detection plane (i.e., $\bar{k}_{\text{BCR}}$) and the component of wavevector along the z direction (i.e., $\hat{z}k_z$). To be specific, we have the generalized Frank-Tamm formula $\cos\theta_{\text{BCR}} = c/n_{\text{BCR}}v$. The working wavelength is 400 nm in (i), 500 nm (j), 600 nm in (k) and 700 nm in (l). The panels in (i-l) indicate that the generalized Frank-Tamm formula $\cos\theta_{\text{BCR}} = c/n_{\text{BCR}}v$ is insensitive to the working wavelength in a very broad wavelength range through the judicious design of broadband angular filter.

Supplementary Note 4: Electromagnetic property of the broadband angular filter for *s*-polarized light

This section discusses about the electromagnetic property of the designed broadband angular filter for the *s*-polarized light. We show in Supplementary Fig. 3 that the designed broadband angular filter in Supplementary Fig. 2 is opaque to the *s*-polarized light with arbitrary incident angle.

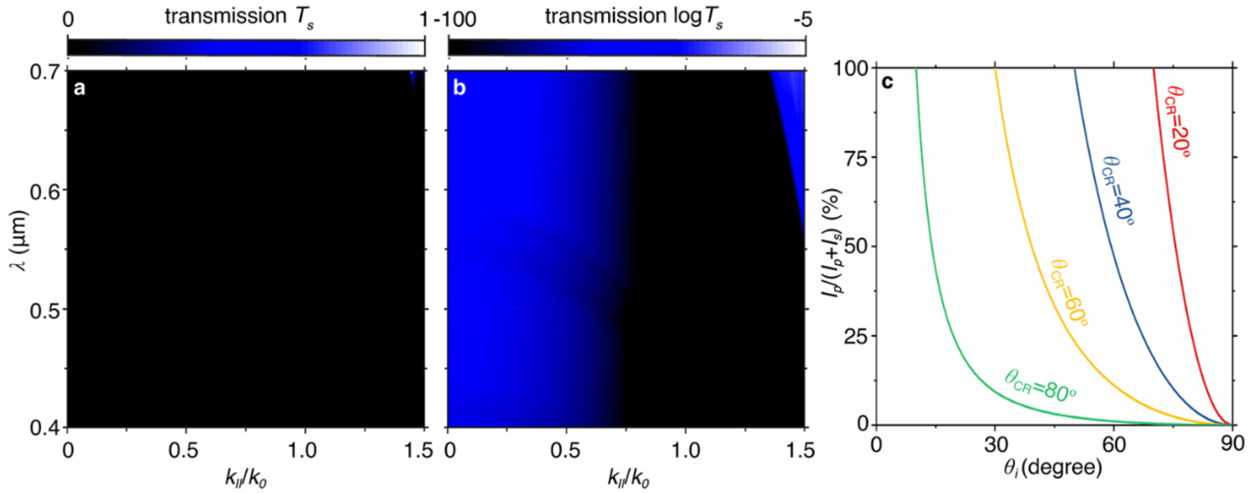

Supplementary Figure 3 | Electromagnetic property of the designed broadband angular filter for the *s*-polarized light. This figure serves as the complementary information for Supplementary Fig. 2. All geometric parameters of the broadband angular filter are the same as those of Supplementary Fig. 2. **a-b**, Transmission of the *s*-polarized light through the designed broadband angular filter. The transmission is plotted in the linear scale in **a**, while for clarity, it is plotted in the logscale in **b**. The transmission of the *s*-polarized light is almost zero (or $< 10^{-5}$) for arbitrary incident angle within the wavelength range of our interest. **b**, Proportion of the *p*-polarized light in the Cherenkov radiation created in the host material. According to the structural setup in Fig. 1, Cherenkov radiation created by the charged particle moving in the host material simultaneously has the *p*-polarized light and the *s*-polarized light. From (a) and Supplementary Fig. 2, only the *p*-polarized component of Cherenkov radiation can safely pass through the broadband angular filter if the incident angle is equal to the Brewster angle. After some calculation, the proportion of the *p*-polarized component in the regular Cherenkov radiation is $\frac{I_p}{I_p+I_s} = \frac{1}{\tan^2\theta_{\mathrm{CR}}\tan^2\theta_{\mathrm{i}}}$, where I_p and I_s are the intensity of the *p*- and *s*-polarized light in the Cherenkov radiation, respectively. In other words, the proportion of *p*-polarized light is dependent on the Cherenkov angle θ_{CR} and the incident angle θ_{i} of light with respect to the broadband angular filter. The *s*-polarized light dominates Cherenkov radiation if the incident angle approaches to 90°.

Supplementary Note 5: Field distribution and photon number of Cherenkov radiation in the detection plane

Field distribution of Cherenkov radiation in the detection plane

After passing through the broadband angular filter, the transmitted Cherenkov radiation in the detection plane in the time domain is shown in Supplementary Fig. 4. From Supplementary Fig. 4, the Cherenkov radiation in the detection plane is symmetric with respect to the projection of the particle trajectory in the xz plane (i.e., the z-axis). We highlight that for Cherenkov radiation passing through the broadband angular filter, while the direction of their in-plane wavevector $\bar{k}_{\mathrm{BCR}} = \hat{x}k_x + \hat{z}k_z$ has a pseudo Brewster-Cherenkov angle with respect to the particle trajectory [Supplementary Fig. 4], their motion in the detection plane is parallel to the particle trajectory (i.e., along the z axis); see their dynamics in the detection plane in the Supplementary Movie 1.

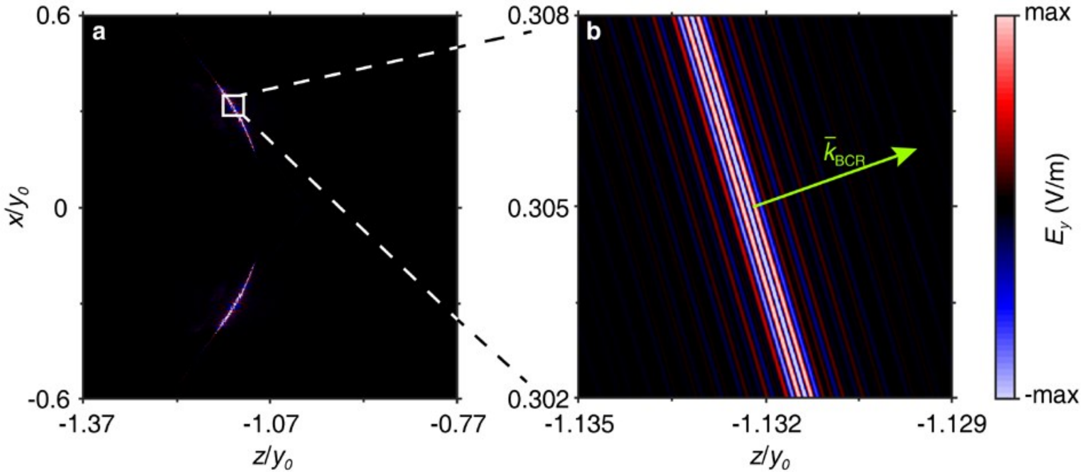

Supplementary Figure 4 | Field distribution of transmitted Cherenkov radiation in the detection plane $y = y_0$ at a specific time moment. For conceptual demonstration, we choose $y_0 = 2.3$ mm and $v = 0.93c$. **a**, Field distribution of E_y. The Cherenkov radiation in the detection plane is symmetric with respect to the z-axis, namely the projection of the particle trajectory in the xz plane. **b**, Enlargement of Cherenkov radiation in (a). From the equiphase wavefront of Cherenkov radiation in the detection plane, it is straightforward to define the in-plane (parallel to the detection plane) wavevector for the transmitted Cherenkov radiation, namely $\bar{k}_{\mathrm{BCR}}$.

Photon number of Cherenkov radiation in the detection plane

In this subsection, we investigate the dependence of the photon number in the detection plane of Brewster-Cherenkov detectors on the structural property of the broadband angular filter. Results are presented in Supplementary Figs. 5-6. Specifically, Supplementary Fig. 5 plots the photon number as a function of wavelength, while Supplementary Fig. 6 shows the photon number as a function of the particle momentum. Different panels present the influence of the detector parameters. These results indicate that the number of Cherenkov photons in the detection plane of Brewster-Cherenkov detectors can be significantly improved through the structural optimization. We compare the photon number to the conventional ring imaging Cherenkov (RICH) detector and show that both achieve the same order of magnitude number of photons.

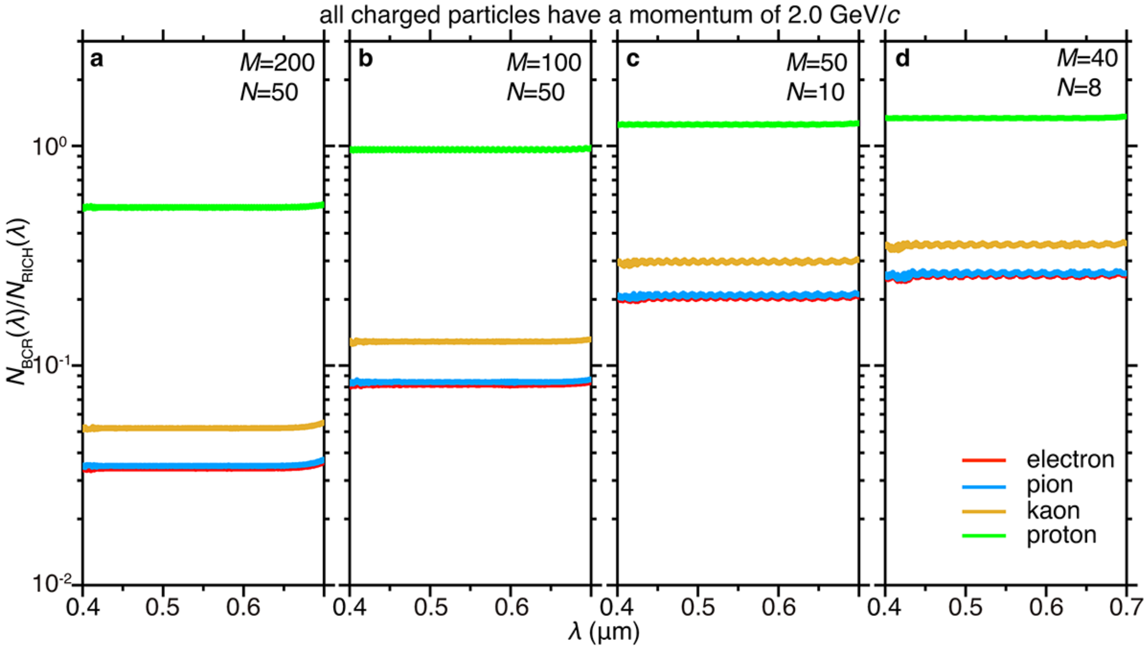

Supplementary Figure 5 | Normalized spectrum of the photon number $N_{\mathrm{BCR}}(\lambda)/N_{\mathrm{RICH}}(\lambda)$. Here, $N_{\mathrm{BCR}}(\lambda)$ ($N_{\mathrm{RICH}}(\lambda)$) is the spectrum of the photon number received in the detection plane of Brewster-Cherenkov detectors (RICH detectors) per unit length of the particle path. For comparison, we set $n = n_{\mathrm{BCR}} = 1.13$, where n_{BCR} is the pseudo refractive index of the broadband angular filter and n is the refractive index of the RICH radiator. The broadband angular filter is constructed by M stacks of 1D photonic crystal with different pitches, and all these 1D photonic crystals have a period number of N.

Different panels correspond to different M and N (with all other parameters the same as those of Fig. 2 in the main text). **a**, $M = 200$ and $N = 50$. **b**, $M = 100$ and $N = 50$. **c**, $M = 50$ and $N = 10$. **d**, $M = 40$ and $N = 8$.

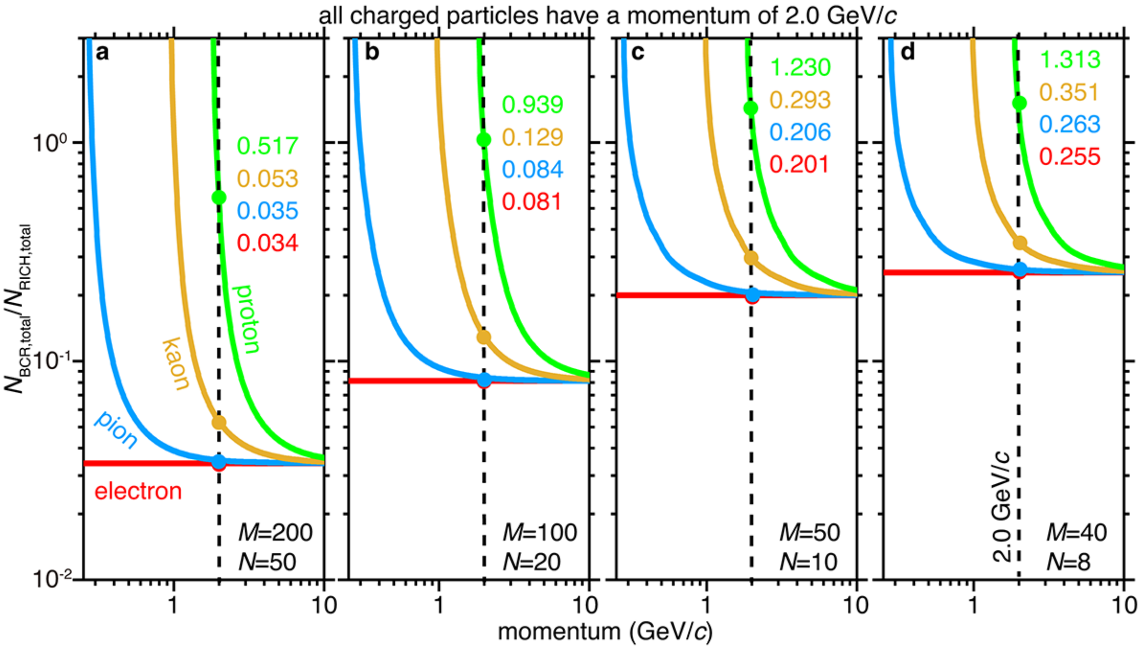

Supplementary Figure 6 | Normalized photon number $N_{\mathrm{BCR}}/N_{\mathrm{RICH}}$ as a function of the momentum for different elementary particles. This figure serves as the complimentary information for Supplementary Fig. 5. $N_{\mathrm{BCR,total}} = \int_{\lambda_{\min}}^{\lambda_{\max}} N_{\mathrm{BCR}}(\lambda)d\lambda$ and $N_{\mathrm{RICH,total}} = \int_{\lambda_{\min}}^{\lambda_{\max}} N_{\mathrm{RICH}}(\lambda)d\lambda$, where $N_{\mathrm{BCR}}(\lambda)$ and $N_{\mathrm{RICH}}(\lambda)$ are given in Supplementary Fig. 5. To perform the integration, we set $\lambda_{\min} = 400$ and $\lambda_{\max} = 700$ nm. Different panels correspond to different particle momenta (with all other parameters the same as those of Fig. 2 in the main text). **a**, $M = 200$ and $N = 50$. **b**, $M = 100$ and $N = 50$. **c**, $M = 50$ and $N = 10$. **d**, $M = 40$ and $N = 8$.

<u>*Width of the intensity distribution of Cherenkov radiation in the detection plane*</u>

The width of the intensity distribution of Cherenkov radiation in the detection plane is plotted in Supplementary Fig. 7, which indicates that the peak-intensity positions of Cherenkov radiation from different charged particles [e.g., kaons and protons] can be well separated from each other.

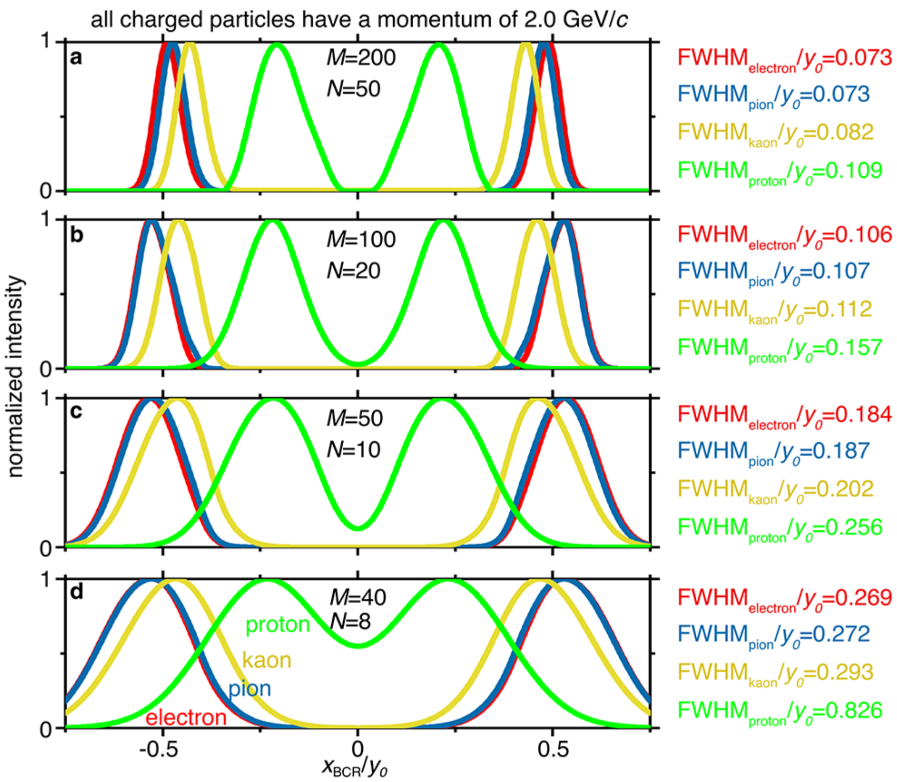

Supplementary Figure 7 | Width of the intensity distribution of Cherenkov radiation in the detection plane. The figure serves as the complimentary information for Supplementary Figs. 5-6. All structural setup here is the same as that in Supplementary Fig. 5. **a-d**, Intensity distribution of Cherenkov radiation in the detection plane with different values of M and N. The full widths at half maxima (FWHM) of the corresponding intensity distribution are given on the right side of each panel.

<u>Influence on the measurement if the charged particles are in close spatial or temporal proximity</u>

Regarding having multiple particles in close spatial or temporal proximity, when the particles themselves do not have strong mutual interactions, they can be treated independently and do not affect the accuracy of measurement. As with other Cherenkov detectors, the multi-particle detection limits then arise from the response time of the photodetectors. Unlike conventional Cherenkov detectors that have a circle attributed for each particle, the Brewster-Cherenkov detector would have two straight lines attributed for each particle. When there are many particles, the images would be many pairs of straight lines in the detection plane. Normally they are well separated (as illustrated below in Supplementary Fig. 8) and therefore easier to

process for particle identification. For example, we plot in Supplementary Fig. 8 the intensity distribution of total Cherenkov photons in the detection plane, when two different charged particles [e.g., a kaon and a proton with the same momentum of 2 GeV/c] travel along the same trajectory and the generated Cherenkov photons in the detection plane from these particles are in close spatial proximity. From Supplementary Fig. 8, the peak-intensity positions for these two charged particles are well separated, and hence they can be used for the identification of these particles. In case there are many such pairs of photon images close to each other in the detection plane, pattern recognition algorithms would need to be developed, which associate particles to pairs of photon images in the detection plane. Such algorithms already exist for RICH and DIRC detectors.

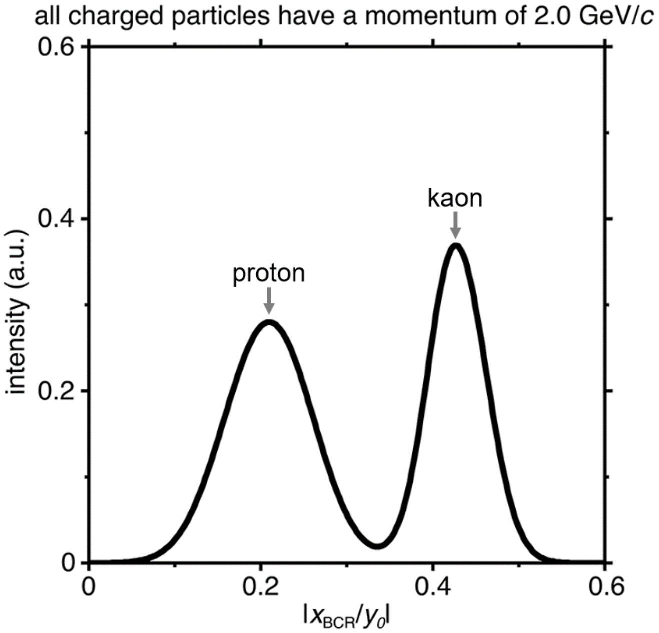

Supplementary Figure 8 | Intensity distribution of total Cherenkov radiation in the detection plane, when a kaon and a proton travel along the same trajectory and the generated Cherenkov photons in the detection plane are in close spatial proximity. Here these charged particles are assumed to have negligible mutual interactions, and they have the same momentum, i.e. 2 GeV/c. This figure serves as the complimentary information for Fig. 2e. All geometric parameters are the same as those of Fig. 2e. As reflected by the plot, the peak-intensity positions for these particles are well separated from each other which is a property useful for the particle identification.

Robustness of the performance of Brewster-Cherenkov detectors with respect to particle's trajectory

From Supplementary Fig. 4 and Fig. 2a-e, the designed Brewster-Cherenkov detector has the potential to infer the projection of the particle trajectory in the xz plane (or the detection plane at $y = y_0$). This is because the intensity distribution of the transmitted Cherenkov radiation in the detection plane is symmetric with respect to the projection of the particle trajectory in the xz plane. Due to this unique feature, the sensitivity of Brewster-Cherenkov detectors is in principle insensitive to the direction of particle velocity, if the particle velocity is parallel to the surface of the broadband angular filter. On the other hand, for the Brewster-Cherenkov detector, the particle trajectory can be very far away from the surface of the broadband angular filter. Then if the particle velocity has a very small angle with respect to the surface of the broadband angular filter (but the particle would not penetrate through the broadband angular filter), the performance of Brewster-Cherenkov detector would not be degraded, since the feature of the transmitted Cherenkov radiation in the detection plane is mostly preserved.

Supplementary Note 6: Peak-intensity position of Cherenkov radiation in the detection plane

Calculation of the peak-intensity position of Cherenkov radiation in the detection plane

In this section, we calculate the peak-intensity position of Cherenkov radiation in the detection plane. Recall that the designed broadband angular filter is comprised of two regular transparent dielectrics. We denote y_a as the total thickness of dielectric slabs with $\varepsilon_{\mathrm{r}1}$; this definition includes the host material (in which the charge particle moves) beneath the particle trajectory and the dielectric region between the bottom surface of the broadband angular filter and the detection plane. Meanwhile, we denote y_b as the total thickness of dielectric slabs with $\varepsilon_{\mathrm{r}2}$. Then the distance between the particle trajectory to the detection plane can be expressed as $y_0 = y_a + y_b$. If the incident angle for the *p*-polarized light is equal to the Brewster angle, the light propagation in dielectric regions with $\varepsilon_{\mathrm{r}1}$ would have a lateral displacement along the x direction as $x_1 = \frac{k_x}{k_{y1}} y_a$. Similarly, the light propagation in dielectric regions with $\varepsilon_{\mathrm{r}2}$ would have a lateral displacement along the x direction as $x_2 = \frac{k_x}{k_{y2}} y_b$. For the light incident with the Brewster angle, we have $k_x =$

$\sqrt{k_{\mathrm{BCR}}^2 - k_z^2}$ in all regions, $k_{y1} = \sqrt{\varepsilon_{\mathrm{r1}} k_0^2 - k_{\mathrm{BCR}}^2}$ in the region with $\varepsilon_{\mathrm{r1}}$, and $k_{y2} = \sqrt{\varepsilon_{\mathrm{r2}} k_0^2 - k_{\mathrm{BCR}}^2}$ in the region with $\varepsilon_{\mathrm{r2}}$, where $k_{\mathrm{BCR}} = n_{\mathrm{BCR}} k_0$. The total lateral displacement experienced by the transmitted Cherenkov radiation can then be expressed as

$$\left|\frac{x_{\mathrm{BCR}}}{y_0}\right| = \left|\frac{x_1+x_2}{y_0}\right| = \frac{\sqrt{n_{\mathrm{BCR}}^2 - \frac{1}{\beta^2}}}{\sqrt{\varepsilon_{\mathrm{r1}} - n_{\mathrm{BCR}}^2}} \frac{y_a}{y_0} + \frac{\sqrt{n_{\mathrm{BCR}}^2 - \frac{1}{\beta^2}}}{\sqrt{\varepsilon_{\mathrm{r2}} - n_{\mathrm{BCR}}^2}} \frac{y_b}{y_0} \quad (5.1)$$

where $\beta = v/c$. Supplementary Figure 9 shows that equation (5.1) can well predict the peak-intensity position of the transmitted Cherenkov radiation in the detection plane.

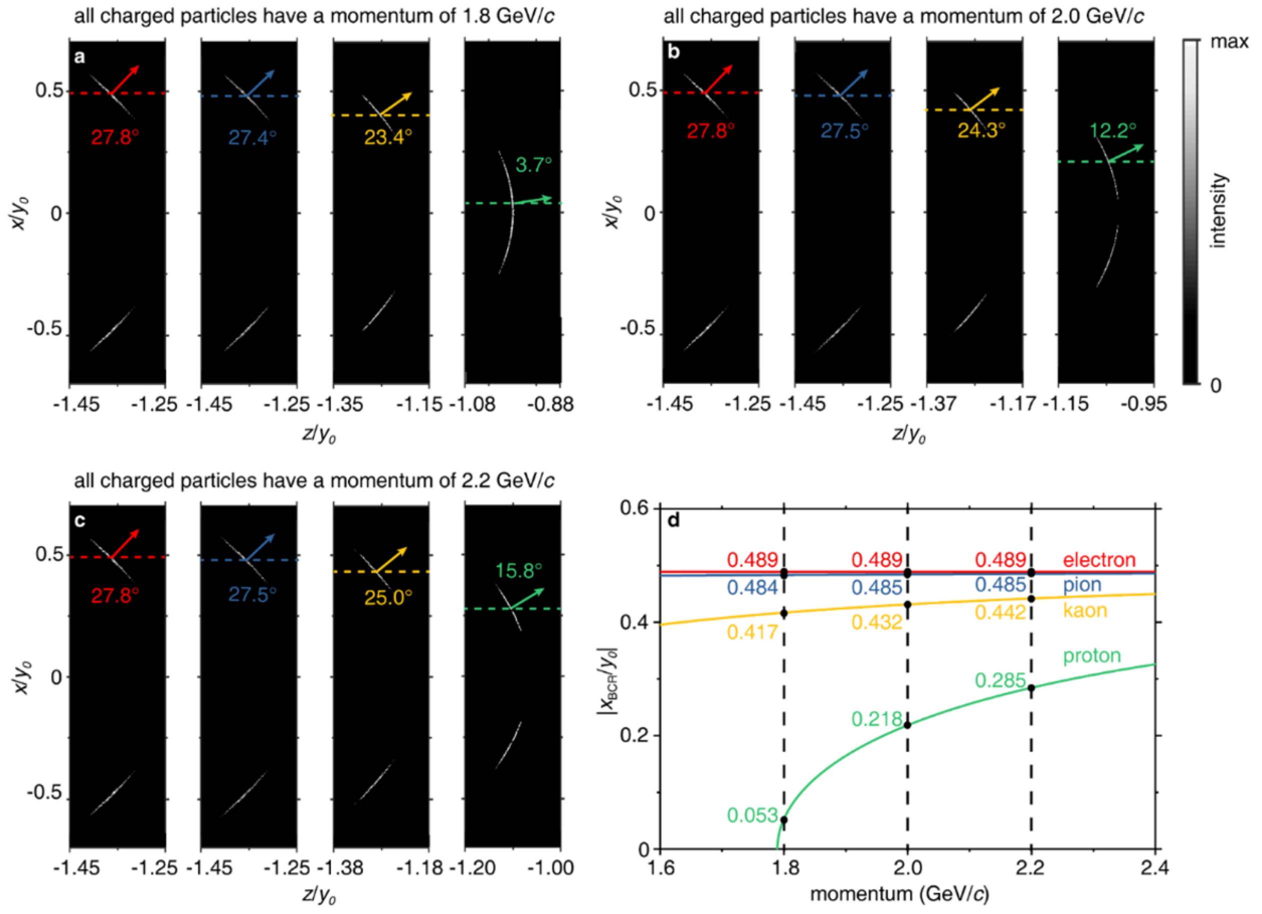

Supplementary Figure S9 | Normalized peak-intensity position of Cherenkov radiation in the detection plane vs the momentum of charged particles. This figure serves as the complementary information for Fig. 4. All the basic structural setups are the same as Fig. 4. The location of the detection

plane is at the plane of $y_0 = 2.3$ mm. **a-c**, Intensity distribution of Cherenkov radiation in the detection plane at some specific time moments for four typical charged particles with different momenta. The momentum for all charged particles is 1.8 GeV/c in (a), 2.0 GeV/c in (b), and 2.2 GeV/c in (c). **d**, Relation between the normalized peak-intensity position $|x_{\mathrm{BCR}}/y_0|$ of Cherenkov radiation in the detection plane and the particle momentum.

Influence of chromatic dispersion in the broadband angular filter on Brewster-Cherenkov detectors

Indeed, all materials have some dispersion. However, we find that for realistic parameters of particle detectors using our Brewster-Cherenkov scheme, the dispersion is sometimes small enough so it does not limit our design. For example, certain transparent dielectrics (e.g., SiO_2 and Al_2O_3 used in this work [44]) have a very small chromatic dispersion in a wide frequency range (e.g., from visible to near-infrared regimes). We now consider the dispersion in our analysis and show in Supplementary Fig. 10 that the chromatic dispersion of these dielectrics in the broadband angular filter will not degrade the performance of Brewster-Cherenkov detectors. Hence, our findings in the main text hold even when including the dispersion in the analysis.

Meanwhile, we highlight in the main text that there are two previously reported proposals for design concepts of nanophotonic Cherenkov detectors. One approach uses metal-based anisotropic metamaterials [24], and the other approach makes use of all-dielectric 1D photonic crystals [25]. Both of these previous nanophotonic Cherenkov detectors can only work in a narrow frequency range, resulting from the inherent large chromatic dispersion of metal in the anisotropic metamaterials [24] or the resonant nature of 1D photonic crystals [25]. In particular, the mechanism proposed in Ref. [25] cannot be applied to achieve broadband Cherenkov detectors even if all constituent dielectrics in 1D photonic crystals are reasonably assumed to be dispersionless. This comparison helps highlight an important advantage of the Brewster-Cherenkov concept we present here: our work for the first time proposes a feasible mechanism to allow the nanophotonic Cherenkov detectors to be designed over a broad frequency range.

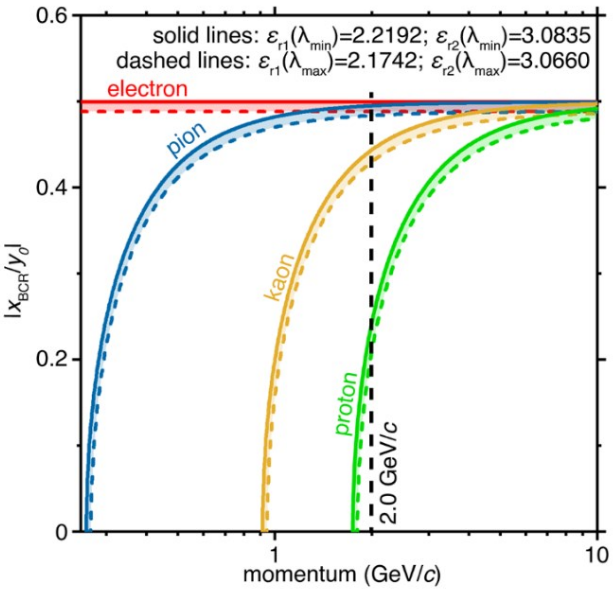

Supplementary Figure 10 | Influence of chromatic dispersion in the two constituent materials of the broadband angular filter on the performance of Brewster-Cherenkov detectors. This figure serves as the complementary information for Fig. 4. The broadband angular filter composed of two regular transparent dielectrics, whose relative permittivities are $\varepsilon_{\mathrm{r1}}$ and $\varepsilon_{\mathrm{r2}}$, respectively. The structural setup is the same as Fig. 4b, except for $\varepsilon_{\mathrm{r1}}$ and $\varepsilon_{\mathrm{r2}}$. In the main text, we set $\varepsilon_{\mathrm{r1}} = 2.18$ (e.g., SiO_2) and $\varepsilon_{\mathrm{r2}} = 3.07$ (Al_2O_3) by neglecting materials' chromatic dispersion. When considering the realistic chromatic dispersion of SiO_2 and Al_2O_3 in the wavelength range of $\lambda_{\mathrm{min}} = 400$ nm to $\lambda_{\mathrm{max}} = 700$ nm, $\varepsilon_{\mathrm{r1}}$ varies from 2.2192 to 2.1742, and $\varepsilon_{\mathrm{r2}}$ varies from 3.0835 to 3.0660 [50,51]. This figure indicates that the small chromatic dispersion in the constituent materials of the angular filter will not degrade the performance of Brewster-Cherenkov detectors.

Influence of the refraction of light through the broadband angular filter on the peak-intensity position of Cherenkov radiation in the detection plane.

If the distance between the particle trajectory and the detection plane y_0 is much larger than the finite thickness of the broadband angular filter, we have $y_a/y_0 \to 1$ and $y_b/y_0 \to 0$. By substituting these approximations into equation (5.1), we have

$$\lim_{y_0\to\infty}\left|\frac{x_{\mathrm{BCR}}}{y_0}\right| = \frac{\sqrt{n_{\mathrm{BCR}}^2-\frac{1}{\beta^2}}}{\sqrt{\varepsilon_{\mathrm{r1}}-n_{\mathrm{BCR}}^2}} = \frac{n_{\mathrm{BCR}}}{\sqrt{\varepsilon_{\mathrm{r1}}-n_{\mathrm{BCR}}^2}}\sin\theta_{\mathrm{BCR}} \quad (5.2)$$

Equation (5.2) indicates the linear relation between $\left|\frac{x_{\mathrm{BCR}}}{y_0}\right|$ and $\sin\theta_{\mathrm{BCR}}$. If θ_{BCR} is small, since $\sin\theta_{\mathrm{BCR}} \approx \theta_{\mathrm{BCR}}$, equation (5.2) also indicates the linear relation between the peak-intensity position of Cherenkov radiation in the detection plane and the pseudo Brewster-Cherenkov angle [Supplementary Fig. 11].

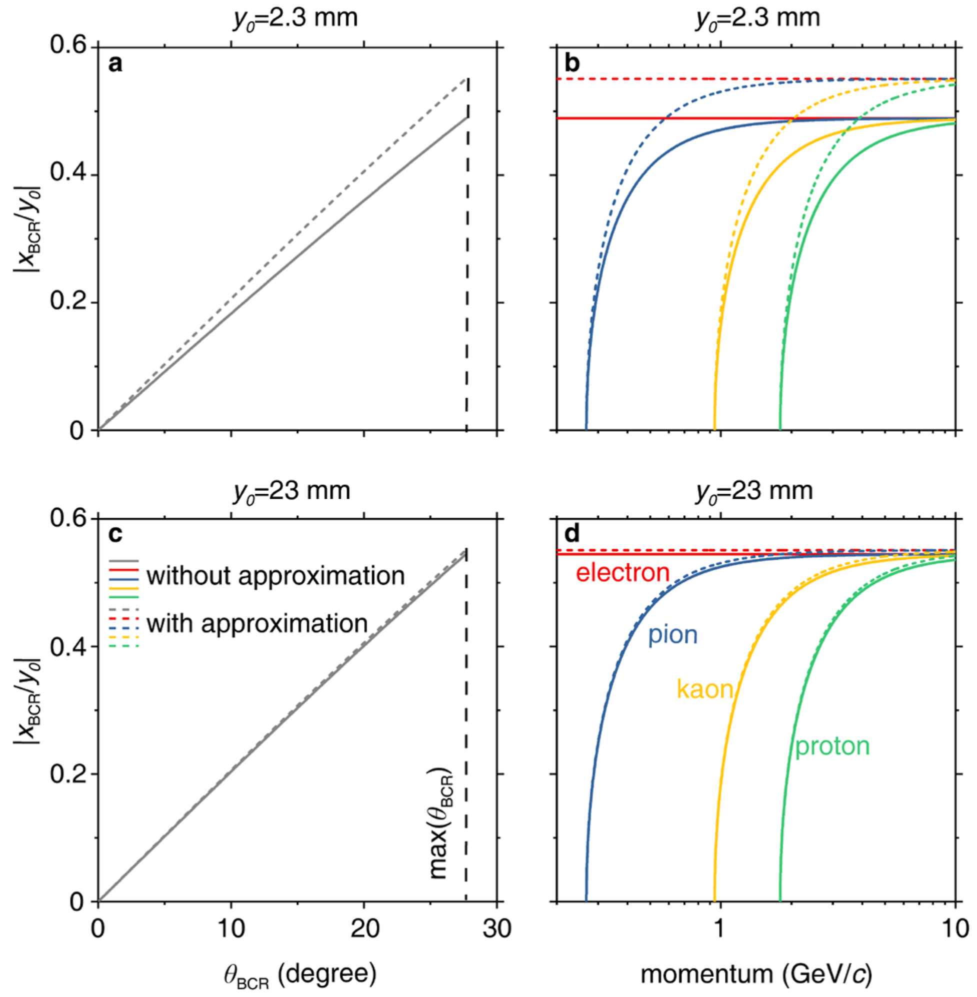

Supplementary Figure 11 | Influence of the refraction of light through the broadband angular filter on the normalized peak-intensity position of Cherenkov radiation in the detection plane. This figure serves as the complementary information for Fig. 4. All the basic structural setups are the same as Fig. 4. The designed broadband angular filter has a total thickness of 2.2 mm. **a**, Normalized peak-intensity

position $|x_{\mathrm{BCR}}/y_0|$ of Cherenkov radiation in the detection plane as a function of the pseudo Brewster-Cherenkov angle θ_{BCR}. **b**, $|x_{\mathrm{BCR}}/y_0|$ as a function of the momentum for four typical charged particles, by applying the relation between the pseudo Brewster-Cherenkov angle and the particle momentum in Fig. 4. The location of detection plane is at the plane of $y_0 = 2.3$ mm in (a, b). **c-d**, $|x_{\mathrm{BCR}}/y_0|$ as a function of the pseudo Brewster-Cherenkov angle or the particle momentum, where the location of detection plane is chosen at the plane of $y_0 = 23$ mm. The dashed lines refer to the approximated results by neglecting the influence of light refraction through the broadband angular filter, namely by setting $\Delta(y_0) = \frac{\Delta x_{\mathrm{BCR}}}{y_0} = 0$. In contrast, the solid lines do not have such an approximation; in other words, they consider the influence of $\Delta(y_0)$ in the calculation of $|x_{\mathrm{BCR}}/y_0|$. For a fixed broadband angular filter, the influence of $\Delta(y_0)$ is negligible if the value of y_0 is large enough, such as the case in (c,d).

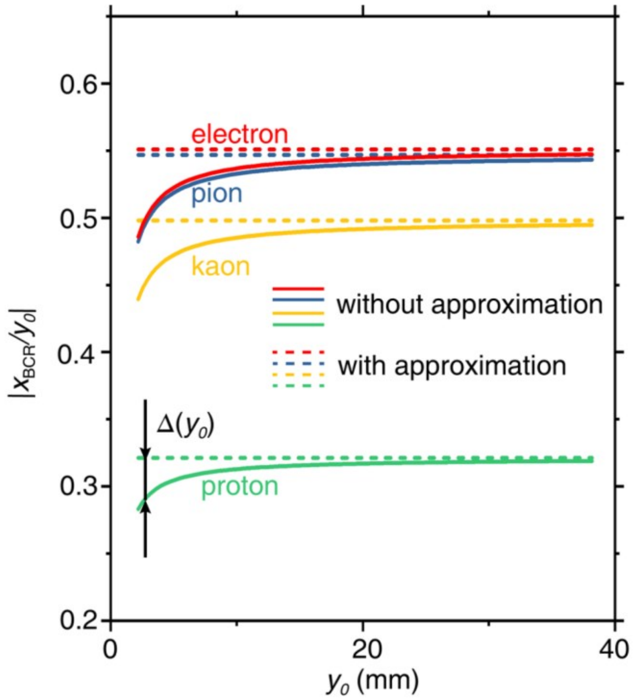

Supplementary Figure 12 | Normalized peak-intensity position $|x_{\mathrm{BCR}}/y_0|$ of Cherenkov radiation in the detection plane for a fixed broadband angular filter as a function of the distance between the particle trajectory and the detection plane, namely y_0. This figure serves as the complementary information for Fig. 4. All the basic structural setup are the same as Fig. 4. The total thickness of the

designed broadband angular filter is 2.2 mm. The dashed lines refer to the approximated results by neglecting the influence of light refraction through the broadband angular filter, namely by setting $\Delta(y_0) = \frac{\Delta x_{\mathrm{BCR}}}{y_0} = 0$. In contrast, the solid lines consider the influence of $\Delta(y_0)$. This figure shows that the influence of $\Delta(y_0)$ is negligible only if the value of y_0 is large enough.

For a finite value of y_0, equation (5.1) is equivalent to $\left|\frac{x_{\mathrm{BCR}}}{y_0}\right| = \frac{n_{\mathrm{BCR}} \cdot \sin\theta_{\mathrm{BCR}}}{\sqrt{\varepsilon_{\mathrm{r1}} - n_{\mathrm{BCR}}^2}} + \Delta(y_0)$, where $\Delta(y_0)$ is the normalized displacement induced by the light propagation inside the dielectric regions with $\varepsilon_{\mathrm{r2}}$ of the broadband angular filter. Based on equation (5.1) and equation (5.2), mathematically, we have

$$\Delta(y_0) = \frac{\Delta x_{\mathrm{BCR}}}{y_0} = -\left(\frac{\sqrt{n_{\mathrm{BCR}}^2 - \frac{1}{\beta^2}}}{\sqrt{\varepsilon_{r1} - n_{\mathrm{BCR}}^2}} - \frac{\sqrt{n_{\mathrm{BCR}}^2 - \frac{1}{\beta^2}}}{\sqrt{\varepsilon_{r2} - n_{\mathrm{BCR}}^2}}\right)\frac{y_b}{y_0} \tag{5.3}$$

The influence of $\Delta(y_0)$ on the value of $\left|\frac{x_{\mathrm{BCR}}}{y_0}\right|$ is negligible only when y_0 is large enough [Supplementary Figs. 11-12].

<u>*Performance of Brewster-Cherenkov detectors when the particle trajectory is not parallel to the top surface of broadband angular filter*</u>

If the particle trajectory is tilted from the parallel plane of the top surface of the broadband angular filter with an angle α, the expression for $\left|\frac{x_{\mathrm{BCR}}}{y_0}\right|$ in equation (5.1) should be modified accordingly. By following a similar calculation procedure of equation (5.1), we obtain $\left|\frac{x_{\mathrm{BCR}}}{y_0}\right| = \frac{k_x}{\sqrt{\varepsilon_{\mathrm{r1}} - n_{\mathrm{BCR}}^2}}\frac{y_a}{y_0} + \frac{k_x}{\sqrt{\varepsilon_{\mathrm{r2}} - n_{\mathrm{BCR}}^2}}\frac{y_b}{y_0}$, where $k_x^2 + \left[\left(\frac{\omega}{v}\right)\cos\alpha - \sqrt{\varepsilon_{\mathrm{r1}}k_0^2 - k_x^2 - \left(\frac{\omega}{v}\right)^2}\sin\alpha\right]^2 = k_{\mathrm{BCR}}^2$. We quantitatively show in Supplementary Fig. 13 that our designed Brewster-Cherenkov detectors can still work well if α is small. For example, if α varies from -0.5^o to 0.5^o, the performance of our Brewster-Cherenkov detector remains almost unchanged [Supplementary Fig. 13].

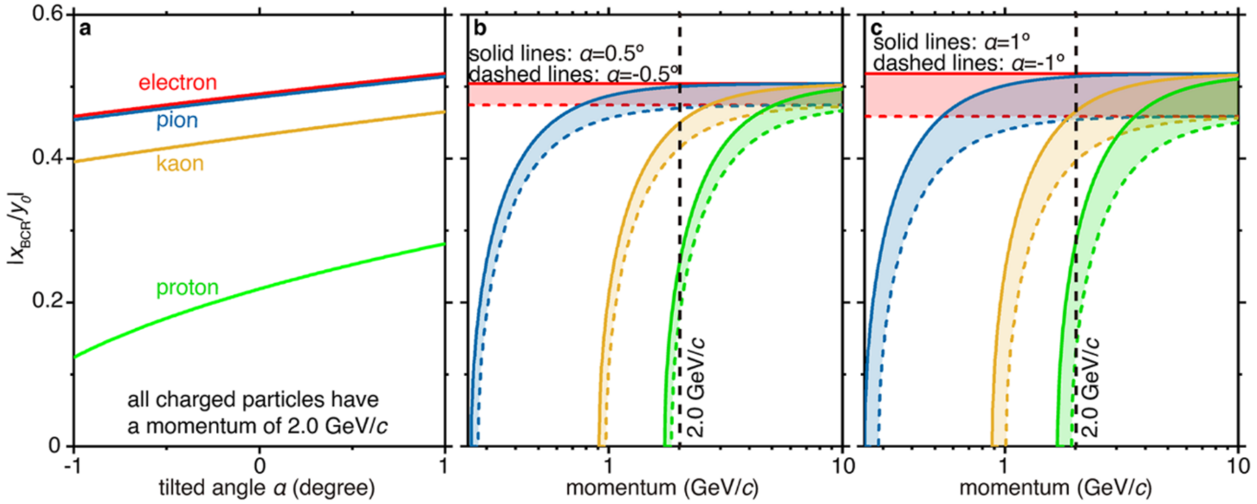

Supplementary Figure 13 | Performance of Brewster-Cherenkov detectors when the particle trajectory has a tilted angle α with respect to the top surface of the broadband angular filter. This figure serves as the complementary information for Fig. 4. All parameters (except for the value of α) are the same as those in Fig. 4b. **a**, Normalized peak-intensity position $|x_{\mathrm{BCR}}/y_0|$ of the transmitted Cherenkov radiation in the detection plane versus the titled angle α. **b-c**, $|x_{\mathrm{BCR}}/y_0|$ as a function of the particle momentum, if α varies from -0.5^o to 0.5^o in (b) or varies from -1° to 1° in (c). This figure indicates that the titled angle within the range of -0.5^o to 0.5^o has a small influence on the performance of Brewster-Cherenkov detectors.

<u>Supplementary Note 7: More discussions on the performance of Brewster Cherenkov detectors</u>

<u>Influence of the pseudo refractive index on the sensitivity and momentum coverage of Brewster-Cherenkov detectors</u>

Supplementary Fig. 14 shows that the pseudo Brewster-Cherenkov angle θ_{BCR} is most sensitive to the particle velocity v around the pseudo Cherenkov threshold. The pseudo Cherenkov threshold is defined as $v_{\mathrm{th,BCR}} = c/n_{\mathrm{BCR}}$, according to the generalized Frank-Tamm formula $\cos\theta_{\mathrm{BCR}} = c/n_{\mathrm{BCR}}v$. Therefore, the pseudo refractive index determines the momentum coverage of Brewster-Cherenkov detectors with high sensitivity.

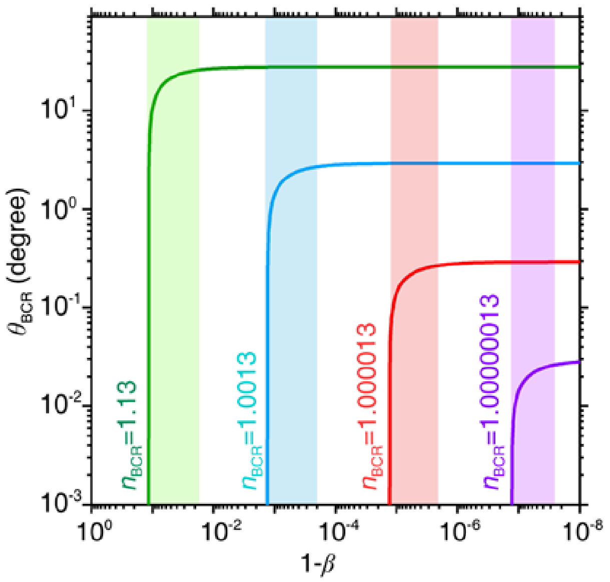

Supplementary Figure 14 | Pseudo Brewster-Cherenkov angle θ_{BCR} as a function of $1 - v/c$ under different values of pseudo refractive index n_{BCR}. Here we denote $\beta = v/c$. This figure is plotted according to the generalized Frank-Tamm formula, namely $\cos\theta_{\mathrm{BCR}} = c/n_{\mathrm{BCR}}v$. Due to the sensitivity of the pseudo Brewster-Cherenkov angle to the particle velocity in the colored regions, these colored regions indicate that the designed Brewster-Cherenkov detectors can have high sensitivity. In other words, Brewster-Cherenkov detectors in principle can work in any desired momentum range with high sensitivity through the judicious engineering of the pseudo refractive index via the Brewster effect.

Influence of the finite thickness of the angular filter on the performance of Brewster-Cherenkov detectors

When the stack number M of 1D photonic crystals and the periodicity number N of each 1D photonic crystal are finite, the p-polarized light incident at the angles very close to the Brewster angle can also safely pass through the broadband angular filter [Supplementary Fig. 15]. This way, there is a small angular (and thus spatial) spread of the transmitted Cherenkov radiation in the detection plane, such as those shown in Fig. 2a-e. This phenomenon would to some extent degrade the sensitivity of Brewster-Cherenkov detectors. However, the sensitivity of Brewster-Cherenkov detector can still be guaranteed by effectively avoiding

this phenomenon, through increasing both the values of M and N in the practical implementation, as shown in Supplementary Fig. 15.

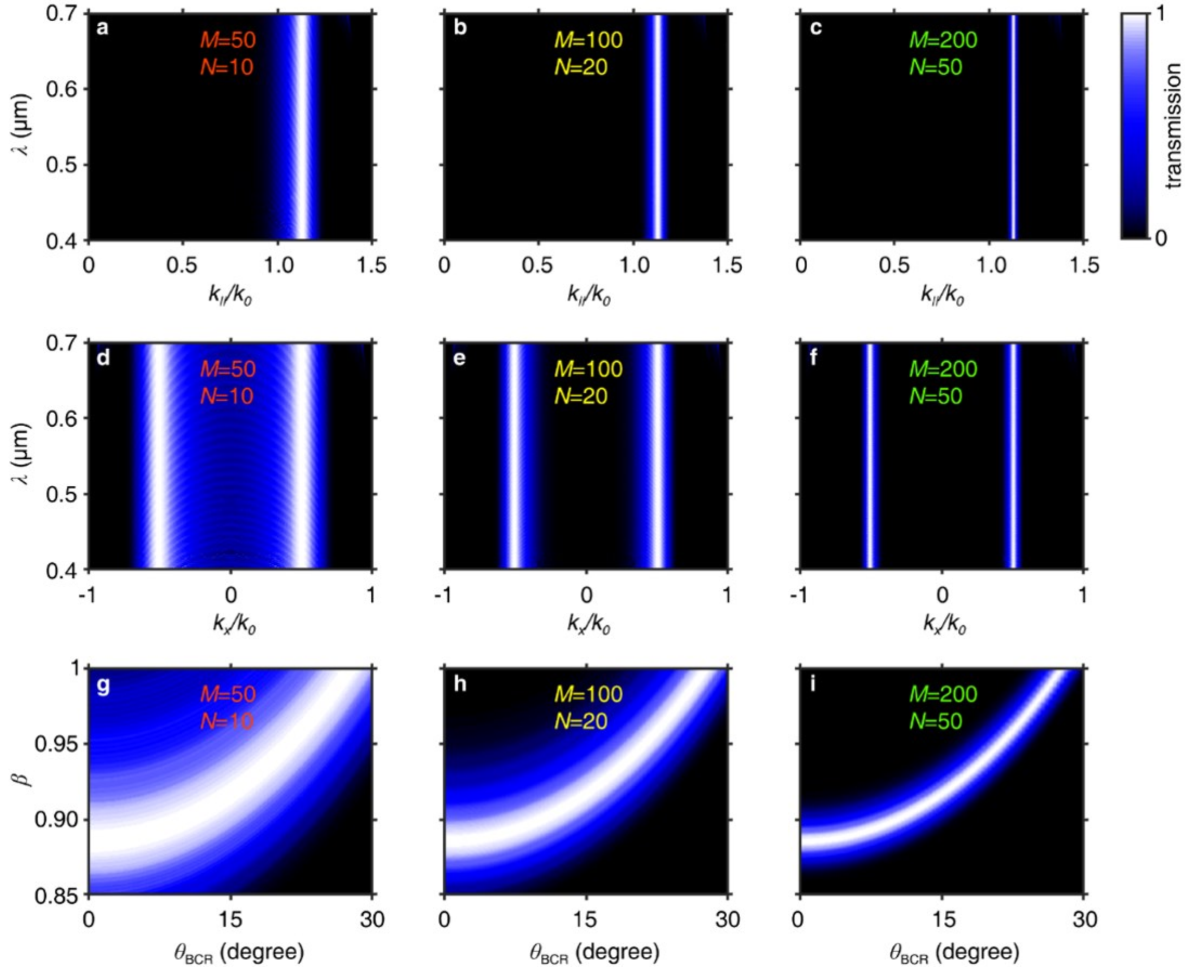

Supplementary Figure 15 | Influence of the finite thickness of the broadband angular filter on the performance of Brewster-Cherenkov detectors. This figure discusses about the p-polarized light. Recall that the broadband angular filter is constructed by M stacks of 1D photonic crystal, and all these 1D photonic crystals have a period number of N. The value of M and N are indicated in each panel. To facilitate the numerical calculation of the field distribution of Cherenkov radiation in the detection plane, we choose $M = 200$ and $N = 50$ in Figs. 2a-e & 4 in the main text. **a-c**, Transmission of light through the broadband angular filter as a function of the wavelength and the in-plane wavevector $k_{||}$. **d-f**, Transmission as a function of the wavelength and the wavevector component k_x under a fixed value of $k_z/(\omega/c)$. The

value of k_z corresponds to the particle velocity, namely $k_z = \omega/v$. Here we choose $\beta = v/c = 0.99$ in (d-f). **g-i**, Transmission as a function of β and the pseudo Brewster-Cherenkov angle θ_{BCR} under a fixed working wavelength of $\lambda = 500$ nm.

Potential choices of the host material where the charged particle moves for Brewster-Cherenkov detectors

We consider in Fig. 1 that the charged particle moves in a homogeneous dielectric material, which is above the broadband angular filter. The relative permittivity of this host material is chosen to be ε_{h} in the main text (e.g., $\varepsilon_{\mathrm{h}} = \varepsilon_{\mathrm{r1}}$ used in the numerical calculation). We highlight that the designed Brewster-Cherenkov detector actually does not have any strict requirements on this host material, because the sensitivity of Brewster-Cherenkov detectors is determined by n_{BCR} of the broadband angular filter, instead of the refractive index of the host material. For example, this host material can be a transparent dielectric with a high refractive index. Note that the photon yield of Cherenkov radiation per unit length along the particle trajectory would increase if the host material has a larger refractive index. Correspondingly, the charged particle moves in the host material with a larger refractive index would have a larger photon yield in the detection plane. Moreover, the Brewster-Cherenkov detector can maintain a good performance when the swift particle moves at a very large distance away from the top surface of the broadband angular filter. Such a particular setup would effectively prevent the direct interaction between the charged particles and the broadband angular filter, and therefore, it can keep a low rate of secondary particle production triggered by the original particle. These feature is useful for Cherenkov detectors in multi-particle environments.

Influence of chromatic dispersion in the Cherenkov radiator medium on the performance of Brewster-Cherenkov detectors

Supplementary Fig. 16 quantitatively shows that the measurement resolution of our Brewster-Cherenkov detectors will not be degraded by a small chromatic dispersion in the transparent radiator medium, e.g. $\pm 2\%$ variation in radiator's permittivity. Such small dispersion is available in existing solid materials.

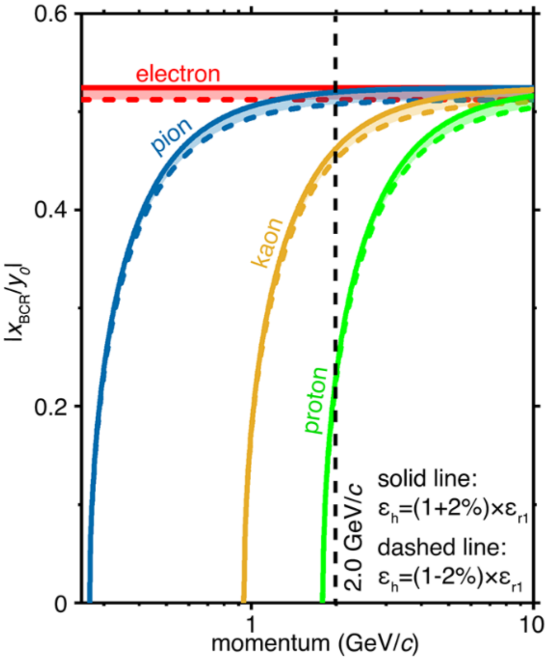

Supplementary Figure 16 | Influence of chromatic dispersion in the radiator medium on the performance of Brewster-Cherenkov detectors. This figure serves as the complementary information for Fig. 4. Here we set the vertical distance between the particle trajectory and the top surface of the broadband angular filter to be 2 mm. All other parameters (except for the permittivity ε_{h}, namely the dielectric constant of the host material in which the charged particle travels)) are the same as those in Fig. 4b. In the main text, we use $\varepsilon_{\mathrm{h}} = \varepsilon_{\mathrm{r1}}$ for the calculation. To illustrate the effect of chromatic dispersion, we set that ε_{h} varies within the range from $0.98\varepsilon_{\mathrm{r1}}$ to $1.02\varepsilon_{\mathrm{r1}}$ in this figure.

<u>Possible experimental realization of Brewster-Cherenkov detectors with the combination of the Cherenkov radiator, the broadband angular filter, and photodetectors</u>

To facilitate future experiments, we schematically show the possible experimental realization of the Brewster-Cherenkov detector in Supplementary Fig. 17.

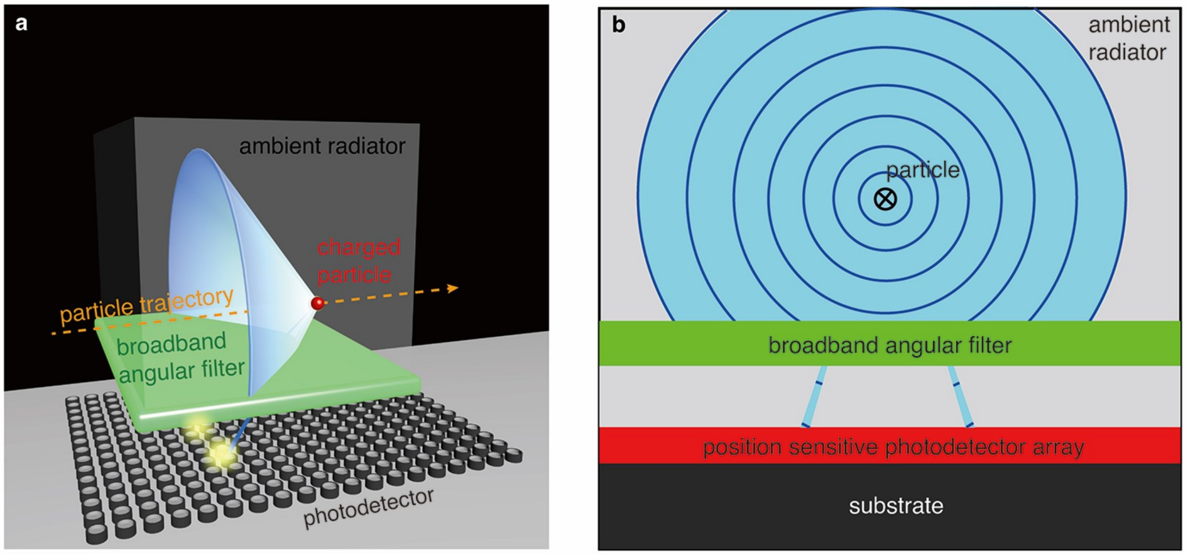

Supplementary Figure 17 | Schematic of Brewster-Cherenkov detectors with the combination of the Cherenkov radiator, the broadband angular filter, and photodetectors. a, 3D view. **b**, side view.
