# Supplementary material for: A Brewster route to Cherenkov detectors
Source: Nat Commun. 2021 Sep 21;12:5554. doi: 10.1038/s41467-021-25822-x (PMC8455627; doi:10.1038/s41467-021-25822-x)
Supplement: Supplementary file 2 — Description of Additional Supplementary Files [file 41467_2021_25822_MOESM2_ESM.pdf]

Description of Additional Supplementary Files

File name: Supplementary Movie 1
Description: Real-space movie of Cherenkov radiation in the detection plane. Here we show the dynamics of the intensity distribution of Cherenkov radiation in the detection plane for a high-energy electron with a velocity of 0.93c. The other structural setups are the same as Figs. 1&2a-e
